# Replacement of the Cobalt Center of Vitamin B_12_ by Nickel: Nibalamin and Nibyric Acid Prepared from Metal‐Free B_12_ Ligands Hydrogenobalamin and Hydrogenobyric Acid

**DOI:** 10.1002/anie.202008407

**Published:** 2020-09-02

**Authors:** Christoph Kieninger, Klaus Wurst, Maren Podewitz, Maria Stanley, Evelyne Deery, Andrew D. Lawrence, Klaus R. Liedl, Martin J. Warren, Bernhard Kräutler

**Affiliations:** ^1^ Institute of Organic Chemistry University of Innsbruck 6020 Innsbruck Austria; ^2^ Center for Molecular Biosciences (CMBI) University of Innsbruck 6020 Innsbruck Austria; ^3^ Institute of General Inorganic and Theoretical Chemistry University of Innsbruck 6020 Innsbruck Austria; ^4^ School of Biosciences University of Kent Canterbury CT2 7NJ UK; ^5^ Quadram Institute Bioscience Norwich Science Park Norwich NR4 7UQ UK

**Keywords:** cobalamins, porphyrinoids, transition metals, crystal structures, vitamins

## Abstract

The (formal) replacement of Co in cobalamin (**Cbl**) by Ni^II^ generates nibalamin (**Nibl**), a new transition‐metal analogue of vitamin B_12_. Described here is **Nibl**, synthesized by incorporation of a Ni^II^ ion into the metal‐free B_12_ ligand hydrogenobalamin (**Hbl**), itself prepared from hydrogenobyric acid (**Hby**). The related Ni^II^ corrin nibyric acid (**Niby**) was similarly synthesized from **Hby**, the metal‐free cobyric acid ligand. The solution structures of **Hbl**, and **Niby** and **Nibl**, were characterized by spectroscopic studies. **Hbl** features two inner protons bound at N2 and N4 of the corrin ligand, as discovered in **Hby**. X‐ray analysis of **Niby** shows the structural adaptation of the corrin ligand to Ni^II^ ions and the coordination behavior of Ni^II^. The diamagnetic **Niby** and **Nibl**, and corresponding isoelectronic Co^I^ corrins, were deduced to be isostructural. **Nibl** is a structural mimic of four‐coordinate base‐off **Cbls**, as verified by its ability to act as a strong inhibitor of bacterial adenosyltransferase.

## Graphical abstract

Nibalamin (**Nibl**), the novel Ni^II^ analogue of vitamin B_12_, was synthesized from the metal‐free B_12_ ligand hydrogenobalamin, prepared from biosynthetic hydrogenobyric acid (**Hby**). An X‐ray crystal analysis of nibyric acid, the Ni^II^ complex of **Hby**, revealed the first structural details of a nickel complex of a natural corrin ligand. **Nibl** is a structural mimic of the corresponding four‐coordinate cobalamins, which are of interest in biological investigations.

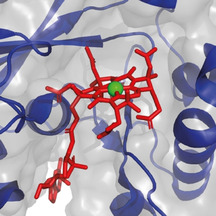

## Introduction

Biologically active vitamin B_12_ derivatives exclusively utilize cobalt as their specific transition metal center, which is bound and activated exquisitely by a helical corrin macrocycle.^[1]^ The metal‐free corrin ligand of vitamin B_12_, hydrogenobyric acid (**Hby**), has recently been made available as a consequence of engineered B_12_ biosynthesis in *E. coli*.[^2^, ^3^] The availability of **Hby** has provided an unparalleled opportunity for the effective synthesis of metal‐free and transition metal analogues of the natural cobalt‐corrinoids a previously intractable challenge in bioinorganic and B_12_ chemistry.^[4]^ We have recently used **Hby** for the synthesis of the corresponding zinc‐corrin zincobyric acid (**Znby**) and the Zn analogue of vitamin B_12_ zincobalamin (**Znbl**), of interest as luminescent structural B_12_ mimics.^[5]^

**Scheme 1:**
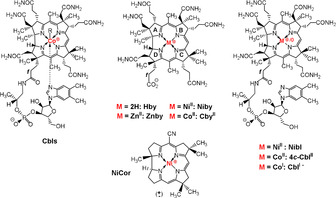
Structural formulae of cobalt, zinc, nickel and metal‐free corrinoids. Left: cobalamins with “base‐on” structures: vitamin B_12_ (R=CN, **CNCbl**), coenzyme B_12_ (*R*=5′‐deoxyadenosyl, **AdoCbl**), methylcobalamin (R=CH_3_, **MeCbl**), cob(II)alamin (R=e^−^, **Cbl^II^**). Center, top: the Ni^II^corrin nibyrate (**Niby**), hydrogenobyric acid (**Hby**), Co^II^‐cobyric acid (**Cby^II^**) and zincobyric acid (**Znby**), where Co^II^ and Zn^II^ carry an unspecified axial ligand (e.g., solvent molecule). Center, bottom: Eschenmoser's synthetic racemic Ni^II^‐corrin **NiCor**.^[6]^ Right: nibalamin (**Nibl**), four‐coordinate “base off” cob(II)alamin (**4** ***c*** **‐Cbl^II^**) and cob(I)alamin (**Cbl^I−^**).

**Scheme 2:**
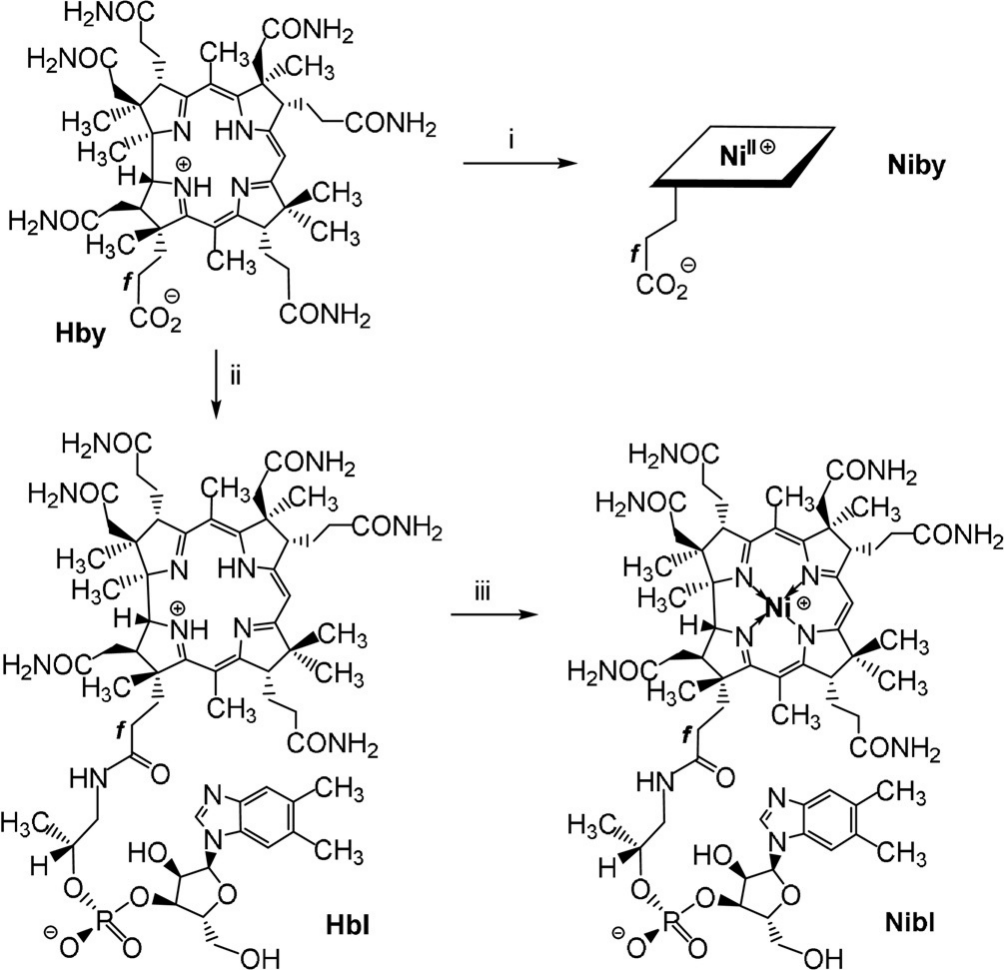
Preparation of the Ni^II^‐corrins **Niby** and **Nibl** from **Hby**. i) 0.5 m Ni(OAc)_2_ pH 6, 1 h, 90 °C, Ar. ii) 3 equiv B_12_ nucleotide moiety,[^1a^, ^12^] HOBt, EDC*HCl, H_2_O, 0 °C, 4 d. iii) 0.5 m Ni(OAc)_2_ pH 6, 1 h, 90 °C, Ar (see the SI for details).

Herein, we report on the first nickel‐complexes of natural corrin ligands, including nibalamin (**Nibl**). We also describe the syntheses of crystalline nibyric acid (**Niby**), the novel Ni^II^ complex of **Hby**,^[3]^ and hydrogenobalamin (**Hbl**), the metal‐free complete B_12_ ligand (see Scheme 1 and Scheme 2). Koppenhagen and co‐workers, back in the 1970’s, reported the isolation of **Hbl** from a *Chromatium* strain supplemented with 5,6‐dimethylbenz‐imidazole (DMB). They were able to characterize **Hbl** by UV/Vis‐spectroscopy and demonstrated that it could be converted into vitamin B_12_ by insertion of cobalt,[^4e^, ^7^] and later reported its mass spectrum.^[8]^

A Ni^II^‐corrin, the **NiCor** (see Scheme 1), was prepared in the Eschenmoser labs as the first synthetic corrin, making use of the Ni^II^ ion as a “template” for the assembly of the corrin macro‐ring.^[6]^ **NiCor** also became the object of the first X‐ray crystallographic investigation of the structure of a non‐cobalt corrin.^[9]^ Four coordinate Ni^II^ complexes prefer to adopt a planar geometry and therefore are more structurally related to the corresponding Co^I^ complexes.^[10]^ Indeed, recently, there has been a resurgence in the quest for close Ni analogues of the B_12_ cofactors.[^4c^, ^4f^] The planar ligand set of **Nibl** potentially represents a structural B_12_ mimic that is inert to the organometallic transformations typical of B_12_‐dependent enzymes, as suggested by its expected coordination chemistry and structural properties. Specific interest in **Nibl**, the Ni^II^‐analogue of vitamin B_12_ and of other cobalamins (**Cbls**) (see Scheme 1), is thus a consequence not only of its chemistry, but also of its possible use as a molecular probe in B_12_ biology and biomedicine, helpful for the investigation of cobalamin‐dependent processes and their physiological effects.^[11]^

## Results and Discussion

**Figure 1:**
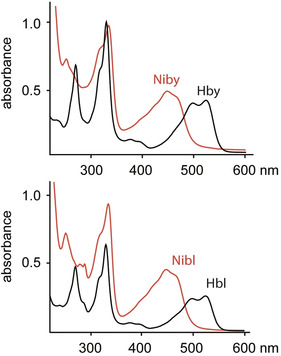
Absorption spectra of aqueous solutions of metal‐free B_12_ ligands **Hby** and **Hbl** and of their Ni^II^‐complexes **Niby** and **Nibl** at 298 K. Top: UV/Vis‐absorption spectra of **Hby** (*c*=31.5 μm, pH 5, black trace) and **Niby** (*c*=34.5 μm, unbuffered, red trace). Bottom: UV/Vis‐absorption of **Hbl** (pH 5, black trace) and of **Nibl** (unbuffered, red trace).

**Figure 2:**
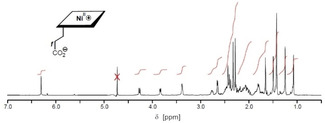
500 MHz ^1^H NMR spectrum of **Niby** in D_2_O (*c*=1.9 mm, 298 K); the water signal after presaturation is marked by an X.

Nibyric acid (**Niby**) was prepared by dissolving 1.40 mg (1.6 μmol) of crystalline hydrogenobyric acid (**Hby**)^[3]^ in 3.5 mL of deoxygenated 0.5 m aqueous Ni^II^acetate, pH 6, with stirring at 90 °C for 75 min. Separation on a short reverse phase column, evaporation and crystallization from aqueous acetonitrile yielded 0.90 mg (0.97 μmol, 61 %) of **Niby**, which was isolated as yellow crystals (see Scheme 2, Exptl. Part and Supporting Information (SI). The UV/Vis absorption spectrum of an aqueous solution of **Niby** displayed bands at 464 nm (shoulder), 448 nm and 334 nm (Figure 1), and exhibited similar gross features to those observed in an absorption spectrum of the Ni‐corrin **NiCor** (but with a slightly red‐shifted maxima).[^6a^, ^6c^] The solution structure of the diamagnetic Ni^II^‐corrin **Niby** (molecular formula C_45_H_64_N_10_O_8_Ni, for HR mass spectra see SI, Figure S3) was analyzed by NMR spectroscopy, providing assignment of all 52 non‐exchangeable H atoms and 44 C atoms (see SI, Figure S4 and Table S1). A 500 MHz ^1^H NMR spectrum of **Niby** in D_2_O displayed five high field singlets for the six methyl groups, a singlet of HC10 at 6.30 ppm, as well as several signals at intermediate field for HC19, HC3, HC8 and HC13 (see Figure 2). The data from homonuclear and heteronuclear correlations confirmed the stereostructure of **Niby** (see SI, Figure S5).

The complete metal‐free ligand of the cobalamins, hydrogenobalamin (**Hbl**), was assembled by attaching the B_12_ nucleotide moiety[^1a^, ^12^] to the propionate moiety of **Hby** at 0 °C through application of the carbodiimide method (Scheme 2).[^4d^, ^13^] In brief, an aqueous solution of 9.12 mg (10.4 μmol) of **Hby** and of 14.71 mg (33.4 μmol) of the B_12_‐nucleotide was treated with 9.4 moleq of HOBt and degassed. To the frozen reaction mixture 4.4 moleq of EDC*HCl were added under Ar. Upon subsequent warm‐up of the reaction mixture to 0 °C, 16 moleq EDC*HCl were added and stirring was continued for 4 d (see SI). Work‐up, using RP18‐chromatographic purification, precipitation with MeCN and drying, furnished 11.3 mg (8.89 μmol, 85 % yield) of **Hbl** as an orange powder. An aqueous solution of **Hbl** at pH 5 exhibited UV/Vis^[4e]^ and CD spectral features (SI, Figure S8 and S9) similar to those of **Hby**.^[3]^ The UV/Vis absorption maximum at 525 nm of the α‐band of **Hbl** and the fluorescence emission maximum at 554 nm (SI, Figure S10) located the first excited singlet state of **Hbl** at *E* ^S^ near 221 kJ mol^−1^, marginally lower than for **Hby**.^[3]^ The structure of **Hbl** (molecular formula C_62_H_90_N_13_O_14_P, see HR‐MS data in SI, Figure S11) in H_2_O was characterized by NMR spectroscopy (600 MHz ^1^H NMR spectrum in SI, Figure S12), providing assignment of 89 H atoms and of all 62 C atoms (see SI, Table S2). The two “inner” H atoms gave rise to singlets at *δ*=12.32 and *δ*=12.57 ppm, which were assigned to H(N4) and to H(N2), respectively, indicating a minor up‐field shift of both of them when compared to **Hby**.^[3]^ The methyl group singlet of H_3_C1 at *δ*=0.81 ppm occurred at 0.47 ppm to higher field, compared to **Hby**, suggesting a temporary residence of the heteroaromatic DMB unit of **Hbl** near to its corrin moiety, a conclusion that was further supported by weak inter‐residual correlations in the ^1^H,^1^H ROESY spectra (see SI, Figure S13). However, the signals of the DMB moiety (HN2 at *δ*=8.35 ppm, HN4 at *δ*=7.31 ppm, HCN7 at *δ*=7.30 ppm) were found at similar chemical shift values to those of the free B_12_ nucleotide,^[12]^ effectively incompatible with a time‐averaged positioning of the DMB part close to the corrin chromophore, as found for zincobalamin (**Znbl**)^[5]^ and for typical “base‐on” Co^III^Cbls.^[14]^

The Ni^II^‐corrin nibalamin (**Nibl**) was prepared by heating a deoxygenated aqueous solution of **Hbl** and Ni(OAc)_2_ for 1 h at 90 °C (Scheme 2), furnishing **Nibl** in 77 % yield as a yellow powder. An unbuffered aqueous solution of **Nibl** exhibited a UV/Vis spectrum that is incompatible with coordination by the DMB base and nearly indistinguishable (at >300 nm) from the spectrum of **Niby**, and similar to the spectrum of the Ni^II^‐corrin **NiCor**[^6a^, ^6c^] (see Figure 1). However, the absorption maxima of **Nibl** occurred at characteristically longer wavelengths when compared to the spectrum of the recently described vitamin B_12_‐derived 5,6‐dihydroxy‐5,6‐dihydronibalamin, which features an interrupted corrin π‐system.^[4f]^ Lower pH values affected only the short wavelength part of the **Nibl** UV/Vis‐absorption spectrum, which was altered by DMB‐protonation, consistent with a p*K*_a_=4.35±0.06 for protonated **Nibl‐H^+^** (see SI, Figure S20).

**Figure 3:**
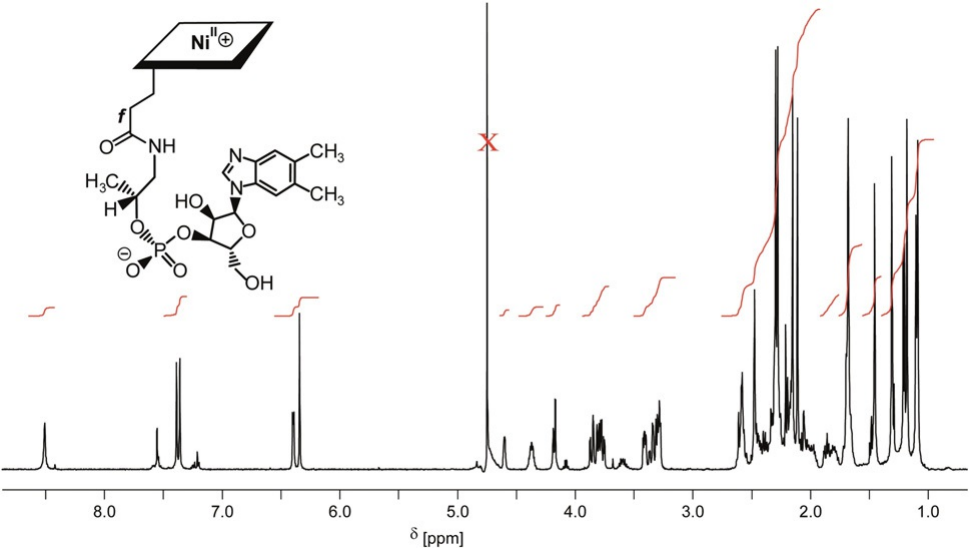
500 MHz ^1^H NMR spectrum of **Nibl** in D_2_O (*c*=1.4 mm, 298 K); residual water signal after presaturation marked by an X.

The structure of **Nibl** (molecular formula C_62_H_88_N_13_O_14_PNi, SI, Figure S17) was characterized in aqueous solution by heteronuclear NMR spectroscopy (see 500 MHz ^1^H NMR spectrum in Figure 3), providing assignment of all 73 non‐exchangeable H atoms and of all 62 C atoms (SI, Table S3). The positions of the singlets of H_3_C1A (*δ*=1.10 ppm), of HCN2 (*δ*=8.51 ppm), HN4 (*δ*=7.36 ppm) and HCN7 (*δ*=7.39 ppm) of the DMB moiety all indicate a base‐off form with a four‐coordinate Ni^II^ center. Hence, the UV/Vis and NMR spectral features of **Nibl** characterize it as an isoelectronic and, roughly, isostructural analogue of the diamagnetic cob(I)alamin (**Cbl^I^**), which is considered to feature a “base‐off” structure with a four‐coordinated Co^I^ center.[^15^, ^16^]

**Figure 4:**
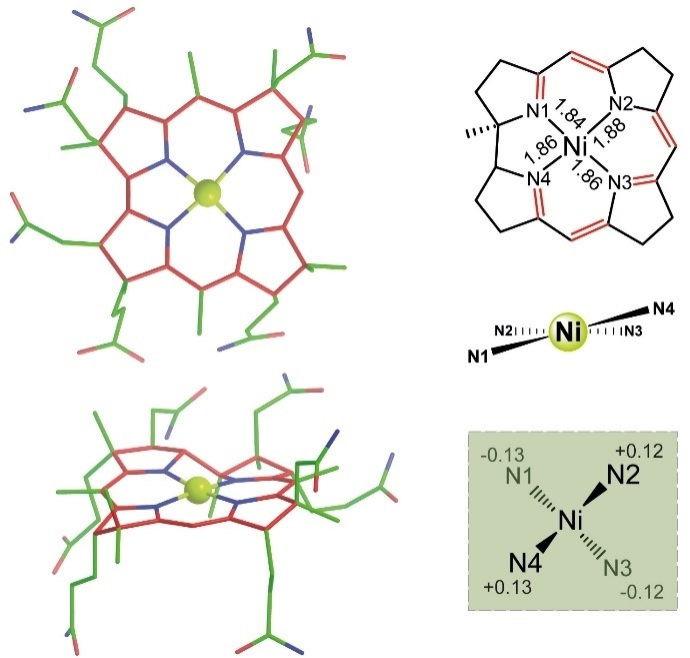
Crystal structure of **Niby**. Left. Crystallographic model of **Niby** in two projections; Right. Graphs representing the corrin core with display of N‐Ni bond lengths (top) and the coordination geometry around Ni^II^ center, highlighting the arrangement of the four inner corrin N atoms in a flattened tetrahedron around the Ni^II^ center (middle and bottom).

The nickel corrin **Niby** was crystallized from aqueous acetonitrile, furnishing yellow single crystals (*P*_2_^1^_2_^1^_2_^1^) suitable for X‐ray analysis (see Figure 4). The incorporation of a Ni^II^ ion into the corrin macrocycle of the metal‐free **Hby** increased the effective symmetry of the corrin ligand as revealed by a comparison of the crystal structures of **Niby** and of **Hby** (see SI for details). Coordination of the Ni^II^‐ion largely equalizes the lengths of the two diagonals, where the N2‐N4 diagonal of **Niby** exceeds its N1‐N3 counterpart by only Δ*d*=0.047 Å, far less than in **Hby** (Δ*d*=0.297 Å)^[3]^ or in **Znby** (Δ*d*=0.197 Å^[5]^). The somewhat longer N2‐N4 diagonals in the metal corrins **Niby** and **Znby** appear to reflect the preferred mode of the conformational adaptation of the coordination hole of the flexible, unsymmetrical corrin ligand to bound metal ions. The radial size of the coordination hole also shrank upon Ni^II^ coordination as the average length of the N1‐N3 and N2‐N4 diagonals of **Hby** (*d*=3.82 Å) was reduced to *d*=3.71 Å in the complex **Niby**. Hence, the coordination of the Ni^II^ ion in **Niby** contracts the corrin ligand and makes it more symmetrical. This latter effect is also expressed by the regularly alternating bond lengths of the corrin π‐system in **Niby**, observations that are compatible with the model Ni‐corrin **NiCor**.^[9]^

The four‐coordinate Ni^II^ ion sits very close to the plane of the four inner corrin N atoms, comparable to the situation in the Ni^II^‐corrin **NiCor**,^[9]^ and in typical Co^III^‐corrins,^[16a]^ but contrasting somewhat with the out of plane distance of 0.048 Å of the five‐coordinate Co^II^ center of heptamethyl‐cob(II)yrinate perchlorate (**Cbin^II^**)^[17]^ (SI, Table S5). As expected,[^9^, ^18^] the metal−N bonds in **Niby** (average Ni−N bond length=1.86 Å) are shorter than those found in the Co^II^ analogue **Cbin^II^** and in the Co^III^‐corrin coenzyme B_12_ (**AdoCbl**), where average (Co^II^‐N) and (Co^III^‐N) bond lengths of 1.89 Å^[19]^ and of 1.90 Å,^[20]^ respectively, were observed.

**Figure 5:**
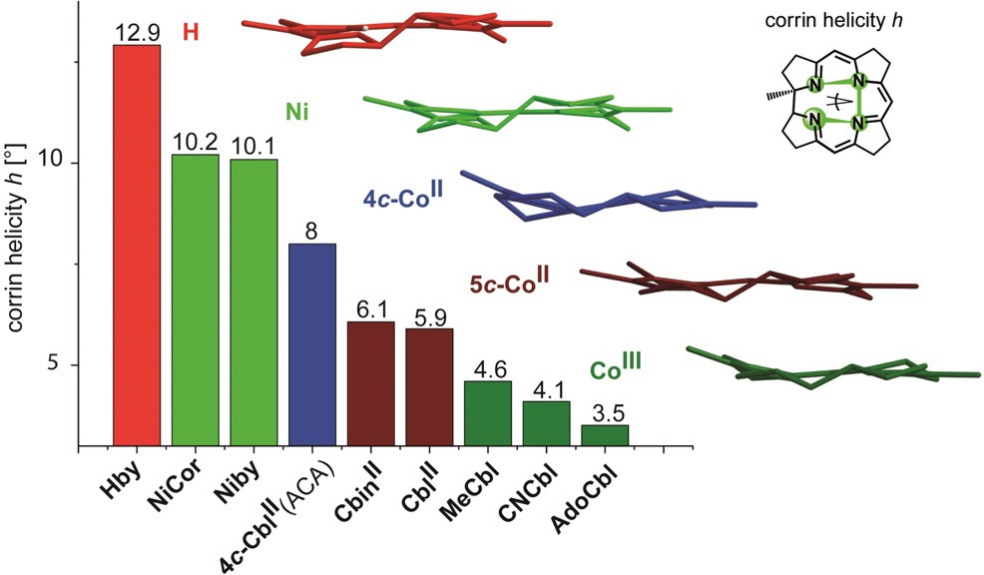
Structural adaptation of the helical corrin ligand to the coordinated metal ions. Comparison of the corrin helicity *h* in the structures of **Hby**, **NiCor**, **Niby**, of **4** ***c*** **‐Cbl^II^** in adenosyltransferase (ACA), of five‐coordinate **Cbin^II^** and **Cbl^II^**, and of six‐coordinate Co^III^‐corrins **MeCbl**, **CNCbl** and **AdoCbl** (see text for details).

**Figure 6:**
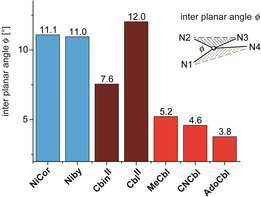
Adaptation of the coordination geometry around the corrin‐bound metal ions to the helical corrin ligand. Comparison of the interplanar angles *φ* in the structures of **NiCor**, **Niby**, 5‐coordinate **Cbin^II^** and **Cbl^II^**, and the six‐coordinate Co^III^ corrins **MeCbl**, **CNCbl** and **AdoCbl** (see text for details).

**Figure 7:**
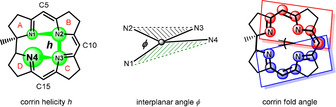
Geometrical parameters describing conformational effects in corrins. Left: corrin helicity (*h*=the dihedral angle N1‐N2‐N3‐N4 around a virtual bond between N2 and N3 of the corrin ligand), revealing the effect of bound metal‐ions on the corrin helix (Figure 5).^[3]^ Center: interplanar angle (*φ*=angle between the two planes through the metal ion and corrin N′s N2/N4 or N1/N4, respectively) characterizing the deviation of the coordination environment at the metal center due to the helical corrin ligand (Figure 6).^[3]^ Right: corrin fold (angle between the best two planes through the inner corrin atoms from N1 to C10 and from C10 to N4, respectively) describing the main conformational response of the corrin macrocycle to the binding of the DMB base in Cbls.^[22]^.

The coordination of the Ni^II^ ion barely affects the conformational properties of the metal‐free corrin ligand (Figures 5,6). Only a slight reduction of the helicity, *h* (Figure 7), of the inner corrin N atoms from *h*=12.9° in **Hby** to *h*=10.1° is seen in **Niby**. Indeed, the effect of the binding of the Ni^II^ ion on the corrin helicity is comparable to the situation in the enzyme‐bound four‐coordinate cob(II)alamin (**4 c‐Cbl^II^**(ACA)) of the adenosyltransferase ACA,^[21]^ for which *h*=8°.^[3]^ In contrast, in five‐coordinate Co^II^‐corrins the corrin helicity is significantly smaller, for example, *h*=6.1° in the Co^II^‐corrin **Cbin^II^**, and in typical Co^III^‐corrins planarization of the corrin ligand is still more pronounced, leading, for example, to *h*=3.5° in **AdoCbl**.^[3]^

The observed lower drive of the four‐coordinate *d* ^8^ Ni^II^ ion to planarize the corrin macrocycle is similarly reflected by its own coordination geometry, which deviates strongly in **Niby** from the coplanar arrangement of the coordinating ligand atoms in typical four‐coordinate low‐spin Ni^II^ complexes.[^9^, ^10^, ^18^] In **Niby** a remarkably large interplanar angle *φ* (Figure 7) at Ni^II^ (*φ*=11.1°) results from extensive directional coordinative adaptation of the Ni^II^‐center to the geometric requirements imposed by the helical corrin ligand (see Figure 6 and SI). *φ* is significantly larger in **Niby** than in Co^III^‐corrins, which exhibit *φ′*s around 5° or less,^[3]^ and is comparable to the situation in five‐coordinate Co^II^‐corrins **Cbl^II^** (*φ*=12°) and **Cbin^II^** (*φ*=7.6°).

The corrin helicity *h* and the inter‐planar angle *φ* (Figure 7), were introduced recently as two complementary parameters characterizing inner conformational effects of the mutual structural adaptation of the corrin macrocycle and of the coordination geometry at the bound metal ion.^[3]^ The so called corrin fold of the helical corrin macrocycle,^[22]^ a classic parameter characterizing the nonplanar corrin ring in Cbls and in other “complete” cobamides (Figure 7), was not used in this current study. Conceived as a measure of the major conformational adaptation of the corrin ring to the cobalt coordination of the (bulky) DMB moiety in “base‐on” Cbls, it runs roughly along the Co‐C10 (east‐west) axis.^[22]^ However, in four‐ and five‐coordinate metal‐corrins lacking the DMB unit, like **Cbin^II^**, **Niby** and **Znby**, the calculated corrin‐fold is dominated by the effects of the corrin helicity and the intersection between the two relevant planes adheres to a north‐south direction (see SI Table S5 and Figure S22).

**Figure 8:**
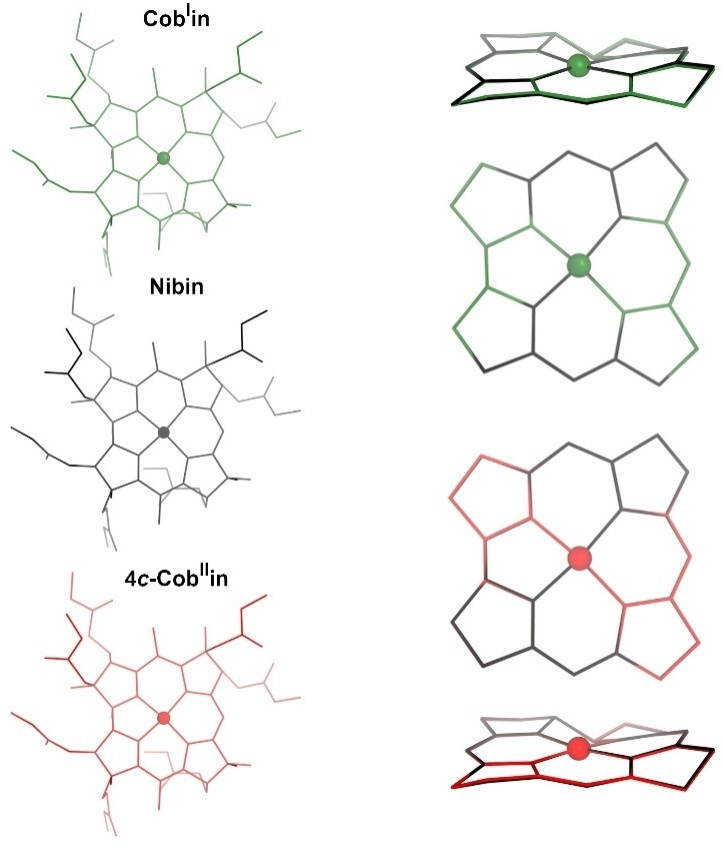
Calculated structures (left) of the four‐coordinate metal‐corrins **Nibin** (black), **Cob^I^in** (green) and **4** ***c*** **‐Cob^II^in** (orange) and structural superpositions (right) of the corrin cores of the cobalt complexes and **Nibin**, minimized at the four coordinating corrin N atoms.

DFT calculations were carried out to test further the proposed close structural similarity between Ni^II^‐corrins and their analogues with four‐coordinate cobalt centers (see Figure 8 and SI for further details). In order to minimize the relevance of peripheral H‐bonds between the amide functions in the implicit solvent calculations, the five‐coordinate lipophilic heptamethyl‐cob(II)yrinate perchlorate (**Cbin^II^**)^[17]^ was used as a starting model. Calculations of the structures of the related unknown corrins heptamethyl‐nibyrinate (**Nibin**), four‐coordinate heptamethyl‐cob(II)yrinate (**4** ***c*** **‐Cob^II^in**) and heptamethyl‐cob(I)yrinate (**Cob^I^in**) were carried out. They indicate extensive structural similarities, but the equatorial metal−N bonds are shorter (by roughly 0.01–0.03 Å) in the Co^I^‐ and Ni^II^‐corrins **Cob^I^in** and **Nibin** in comparison to the four‐coordinate Co^II^‐corrin **4** ***c*** **‐Cob^II^in** and 5‐coordinate **Cbin^II^**. The N1‐N3 diagonal was calculated to be shorter than its N2‐N4 by roughly 0.01–0.02 Å, which is also in good qualitative agreement with the crystallographic data for **Niby** and **Cbin^II^**.^[17]^ All 4‐coordinate metal centers (Ni^II^, Co^I^ and Co^II^) were calculated to be located virtually in the best plane through the four corrin N‐atoms. The latter is arranged in a helix with a calculated value of *h* slightly decreasing from **Nibin** (7.6°) to **Cob^I^in** to **4 c‐Cob^II^in** (6.6°), and induced an interplanar angle *φ* that slightly decreased in the same order (from 8.4° to 7.5°). The calculations for five‐coordinate **Cbin^II^** also reproduced, qualitatively, the still smaller value for *h* (5.4°), a larger value for *φ* (11.3°) and a significant displacement of the Co^II^ center towards the axial β‐ligand (calculated as 0.112 Å).

The deduced utility of **Nibl** as a specific new structural mimic of four‐coordinate base‐off **Cbls** was initially tested in binding studies of **Nibl** to an adenosyltransferase enzyme (ATP:Co^I^rrinoid adenosyltransferase), which catalyzes the biosynthetic construction of **AdoCbl** by Co_β_‐adenosylation of bound **Cbl^I−^**. Such bacterial^[23]^ and human adenosyltransferases,^[24]^ for example, BtuR,^[23a]^ CobA,^[23b]^ ACA,^[21]^ and CblB,^[24]^ have been shown to facilitate the adenosylation process by first inducing the corrin substrate to adopt a four‐coordinate structure, thus raising the redox potential of the Co^II^/Co^I^ couple by around 250 mV,[^23^, ^24^] thereby allowing the physiologically difficult reduction. We, thus, investigated the effect of **Nibl** on the adenosylation process by incubating the *Brucella melitensis* BtuR^[23a]^ and the structural Cbl mimic **Nibl** in the presence of an excess of **Cbl^I^** (see SI, Figures S27–S29). As expected, **Nibl** itself was not a substrate for the enzyme and was not adenosylated. However, the presence of **Nibl** within the incubation did effectively inhibit the adenosyltransferase, reducing the activity of the enzyme by 38 % at a concentration of 1 μm, and by 60 % at 5 μm (Figure S29). Thus, the four‐coordinate Ni^II^ center of **Nibl** affords it the ability to bind within the active site of the adenosyltransferase and to prevent the productive binding of the natural substrate **Cbl**.

## Conclusion

Herein, we have described the first Ni^II^ analogues of natural cobalt corrins vitamin B_12_ (**CNCbl**) and cobyric acid (**Cby**). The new Ni‐corrins **Nibl** and **Niby** display the same basic structural features and lack of coordinative activity as synthetic model Ni^II^‐corrins, such as **NiCor**.[^1a^, ^6a^, ^6c^] The Ni^II^‐corrins are known to be exceptionally stable in regard to the chemical removal of their metal center,^[25]^ and to exhibit no affinity for axially coordinating ligands.[^1a^, ^6c^, ^7^] This latter feature has been rationalized by the extraordinary stabilization of the low‐spin d^8^ configuration by the ligand field of the ring‐contracted corrin ligand.[^1a^, ^9^] As shown here, the natural corrin ligand undergoes only a small contraction of its coordination hole by about 0.03 to 0.04 Å to accommodate the low‐spin Ni^II^ ion. Indeed, the 15‐membered inner perimeter provided by the natural corrin macrocycle, selected for binding low‐spin cobalt ions, also binds Ni^II^ consistently in its low‐spin state. In contrast, in the porphyrinoid B_12_‐related nickel complex coenzyme F_430_[^1a^, ^18^, ^26^] the 16‐membered porphyrinoid macrocycle is a key player in the active, specific adjustment of the spin state and coordinative activity of the nickel center to its function in the enzyme catalyzed methane formation.^[27]^ Indeed, the discovery of coenzyme F_430_ provoked an entirely new look at the structural effect of the tetrapyrrolic macrocycle on the coordination chemistry of bound first‐row transition metals.^[18]^

A common feature of the valence shell of the low spin states of the transition metal ions Ni^II^, Co^III^, Co^II^ and Co^I^ is their unoccupied $d_{x^{2}-y^{2}}$ orbital, a key factor responsible for their strong sigma bonding interactions with the four inner corrin N atoms, leading to similar radial characteristics of their corrin complexes. A differing number of valence shell electrons in Ni^II^‐, Co^III^‐, Co^II^‐ and Co^I^‐corrins is transduced primarily into characteristically different reactivity of the metal centers in the axial direction, strongly affecting their potential binding sites there. Consequently, Ni^II^‐corrins are to be considered particularly well‐suited structural mimics of corresponding isoelectronic Co^I^‐corrins, the critical intermediates in heterolytic organometallic transitions in B_12_‐dependent enzymes.^[28]^ Crystallographic insights and DFT‐based structure calculations also indicate a structural similarity between Ni^II^‐corrins and the exceptional four‐coordinate Co^II^‐corrins. This result contrasts strikingly with the mutually different structures of the typical five‐coordinate Co^II^‐corrins and their Zn^II^‐analogues^[5]^ with similarly sized metal ions^[29]^ that differ by the number of electrons in the valence shell.

The structural analysis of the Nibyrinates predicts that the constitutively robust **Nibl** would likely be an excellent redox‐resistant structural mimic for the elusive cob(I)alamin (**Cbl^I^**), a highly reactive redox‐active intermediate^[30]^ that is found in B_12_‐dependent methyl group transferases, such as methionine synthase,^[28a]^ as well as in the biosynthesis of **AdoCbl** from **Cbl^II^** (via **Cbl^I^**) by Cbl‐adenosyltransferases.[^23^, ^24^] **Nibl** may, likewise, act as a good structural mimic of the recently described natural four‐coordinate Co^II^‐corrins, proposed as key intermediates in the enzymatic transformations catalyzed by the vitamin B_12_ tailoring enzyme CblC,^[31]^ in corrinoid dehalogenases,^[32]^ or as substrates for the reduction to Co^I^‐species in enzymatic cobalt alkylation.[^14^, ^33^] Indeed, as verified here, **Nibl** is a very effective inhibitor of the bacterial Cbl‐adenosyltransferase BtuR.

We have developed a rational and direct synthetic path from hydrogenobyric acid **(Hby**) via hydrogenobalamin (**Hbl**) to nibalamin (**Nibl**), a novel transition‐metal analogue of the **Cbls**. Our recent studies with the Rh^III^ analogue **AdoRhbl** of **AdoCbl**,^[4d]^ with the Zn^II^ analogue **Znbl** of **Cbl^II^**,^[5]^ and now the Ni^II^ analogue **Nibl** of **Cbl^I^**, have furnished a valuable suite of cobalamin mimics for use in the study of B_12_‐dependent enzymatic processes,[^16a^, ^28^, ^30^, ^34^, ^35^] and in B_12_‐dependent biological regulation.^[36]^ Well‐characterized and adequately accessible transition metal analogues (**Metbls**) of the Cbls provide a promising small‐compound platform that may contribute significantly to the ongoing quest for innovative B_12_‐based biological and biomedical applications.[^11^, ^37^] Along these lines, some **Metbls** may find applications as effective antivitamins B_12_.[^4d^, ^11^] The availability of selected **Metbls** and of related metal corrins (**MetCors**) will also allow more detailed experimental investigations into the chemical relevance of the coordination of transition metal ions by the uniquely skewed, strongly helical and unsymmetric natural corrin ligands.^[3]^ Such studies will endow a more informed understanding of the specific evolutionary selection of cobalt rather than any other transition metal^[1]^ for the task of complex organometallic catalysis achieved by the B_12_ cofactors.

## Conflict of interest

The authors declare no conflict of interest.

## Supporting information

As a service to our authors and readers, this journal provides supporting information supplied by the authors. Such materials are peer reviewed and may be re‐organized for online delivery, but are not copy‐edited or typeset. Technical support issues arising from supporting information (other than missing files) should be addressed to the authors.

Supplementary
