# Supplementary material for: Replacement of the Cobalt Center of Vitamin B12 by Nickel: Nibalamin and Nibyric Acid Prepared from Metal‐Free B12 Ligands Hydrogenobalamin and Hydrogenobyric Acid
Source: Angew Chem Int Ed Engl. 2020 Sep 2;59(45):20129–36. doi: 10.1002/anie.202008407 (PMC7693184; doi:10.1002/anie.202008407)
Supplement: Supplementary file 1 — Supplementary [file ANIE-59-20129-s001.pdf]

Angewandte Chemie
Eine Zeitschrift der Gesellschaft Deutscher Chemiker

Supporting Information

Replacement of the Cobalt Center of Vitamin B_{12} by Nickel: Nibalamin and Nibyric Acid Prepared from Metal-Free B_{12} Ligands Hydrogenobalamin and Hydrogenobyric Acid

Christoph Kieninger, Klaus Wurst, Maren Podewitz, Maria Stanley, Evelyne Deery, Andrew D. Lawrence, Klaus R. Liedl, Martin J. Warren, and Bernhard Kräutler**

anie_202008407_sm_miscellaneous_information.pdf

Supporting Information

Table of Contents

1. Materials S2
2. Spectroscopy and instruments S2
3. Synthesis, isolation and spectral analysis of nibyric acid (Niby) S2
4. Synthesis, isolation, and spectral analysis of hydrogenobalamin (Hbl) S8
5. Synthesis, isolation, and spectral analysis of nibalamin (Nibl) S15
6. Determination of the pK_a of protonated Nibl S20
7. X-ray crystal structure analysis of Niby S21
8. Computational studies S25
9. Inhibition studies with Nibl and adenosyltransferase S30
10. References S33

1. Materials

B_{12}-Nucleotide was synthesized according to [1]. Methanol (MeOH), acetonitrile (MeCN), HiPerSolv Chromanorm and acetic acid (HOAc) p.A., sodium hydroxide (NaOH) p.A. from VWR chemicals; nickel acetate trihydrate ($Ni(OAc)_2$*3 H_2O) p.A. from Sigma Aldrich; tetrafluoroboric acid 48% in H_2O from Sigma Aldrich; sodium tetrafluoroborate, p.A., sodium acetate (NaOAc); sodium dihydrogenphosphate (NaH_2PO_4), disodium hydrogenphosphate (Na_2HPO_4), 1-hydroxybezotriazole purum, R(-)-1-amino-2-propanol 98% from Fluka; N-(3-Dimethylaminopropyl)-N′-ethylcarbodiimide hydrochloride (EDC*HCl) 98% from Alfa Aesar; water purified by reversed osmosis and MilliQ academic system; D_2O 99.96%D from Eurisotop; Sep-Pak® C18 cartridges (various sizes, conditioned with 20 mL MeOH and 60 mL H_2O prior to use) from Waters.

2. Spectroscopy and Instruments

UV-Vis: Agilent Cary 60. CD: Jasco J-715 spectropolarimeter or Jasco J-1500-150 CD spectrometer, spectra were recorded at 298K. NMR: 500 MHz Varian Unity Inova, 5mm triple-resonance probe with z-gradients, puls sequences from VNMRJ-ChemPak 4.1; 600 MHz Bruker Avance II+ with Prodigy TCI™ probe; ^1H reference to δ(HDO) = 4.75 ppm, chemical shift and signal assignment was based on ^1H, (^1H,^1H)-COSY, (^1H,^{13}C)-HSQC, (^1H,^{13}C)-HMBC and (^1H,^1H)-ROESY spectra; ESI-HR-MS: Thermo Scientific LTQ-Orbitrap XL, (+)-ion mode, 4.5 kV in MeOH. HPLC: Hitachi Elite LaChrom, L2130 pump, L245 diode array detector; Dionex Ultimate 3000, variable wavelength detector; column: YMC-Triart –C18, 250x4.7 mm, S-5 µm, 12 nm; solvent composition: A: 10 mM aqueous NH_4OAc pH 7, B= MeOH; 8% to 95%B 0-40 min, 95% B 40-44 min, 95% to 8%B 44-45 min, flow= 1 mL/min. RP18-MPLC: Büchi C-605 pump module (binary) flow≈10 mL/min, home-packed RP18 column (l=230 mm, Ø=26 mm, column volume (*cv*) = 122 mL) using about 100 g LiChroprep RP18.

3. Synthesis, isolation and spectral analysis of nibyric acid (Niby)

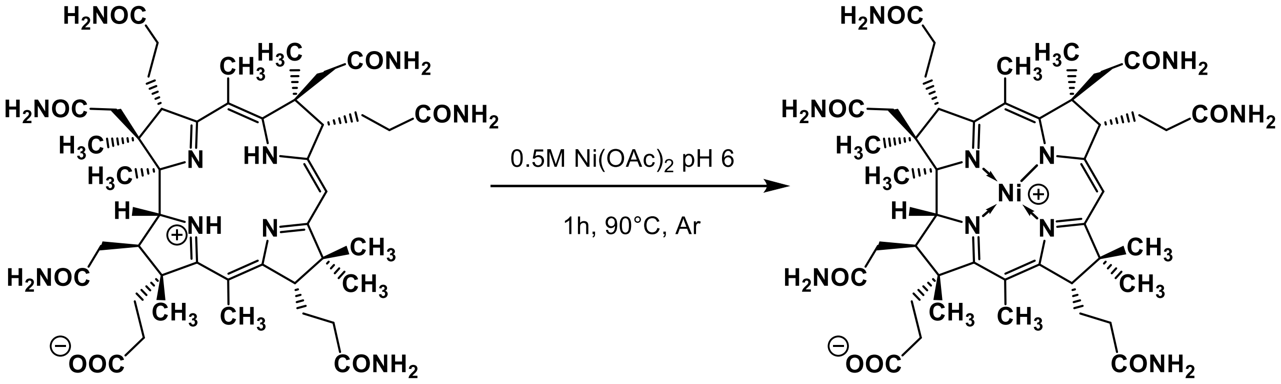

$C_{45}H_{66}N_{10}O_8$
M= 875.09g/mol

$C_{45}H_{64}N_{10}NiO_8$
M= 931.76g/mol

In a 15ml Schlenk tube equipped with a reflux condenser 1.40mg (1.60µmol) cryst. hydrogenobyric acid (**Hby**)[2] were dissolved in 3.5ml 0.5M aqueous $Ni(OAc)_2$ pH 6 and degassed by 7 freeze/vacuum/thaw cycles. The apparatus was pressurized with Ar

and the red-brown mixture was heated to 90 °C for 1h15min. The green solution was cooled on ice for 15min. and diluted with 10ml ice-water. The solution was loaded on a 360mg Sep-Pak cartridge. The adsorbate was washed with 20 ml H_2O, 20 ml 100 mM $NaBF_4$ pH 6 and 20 ml H_2O. The nibyric acid (**Niby)** was eluted with 3ml 100 µM $NaBF_4$ in MeOH and evaporated on the rotary evaporator (40 °C water bath). The yellow residue was dissolved in 10 µl 100 mM $NaBF_4$ pH 6 and 50 µl H_2O and precipitated by the slow addition of ~3 ml MeCN at RT. After resting over night the mother liquor was removed and the **Niby** precipitate was dissolved in a 50µl H_2O and 200 µl MeCN. The **Niby** was crystallized from the yellow solution in dry MeCN atmosphere at RT over 4 days. The crystals were separated from the ML and dried in HV overnight. 0.90 mg (0.97 µmol, 61%) crystalline nibyric acid (**Niby**) were obtained.

UV/Vis (c=34.5 µM in H_2O): λ^{max} (lg ε) = 464 (sh., 4.10), 448 (4.13), 425 (sh., 4.02), 403 (sh., 3.91), 334 (4.44), 321 (sh., 4.36), 252 (4.31),

CD (46.0 µM in H_2O): $\lambda^{max/min}$ (±Δε [l*mol^{-1}*cm^{-1}]) = 460 (-5.2), 413 (2.8), 350 (-2.9), 327 (sh., 21.4), 314 (28.3), 277 (sh., -3.1), 256 (-29.9); λ^0 = 430, 391, 344, 288, 226

HR-ESI-MS (MeOH): m/z (%) = 935.388 (10), 934.430 (27), 933.427 (30), 932.426 (76), 931.432 (100, $[C_{45}H_{64}N_{10}NiO_8+H]^+$ ≙ $[M+H]^+$); 477.713 (17), 477.211 (23, $[M+H+Na]^{2+}$)

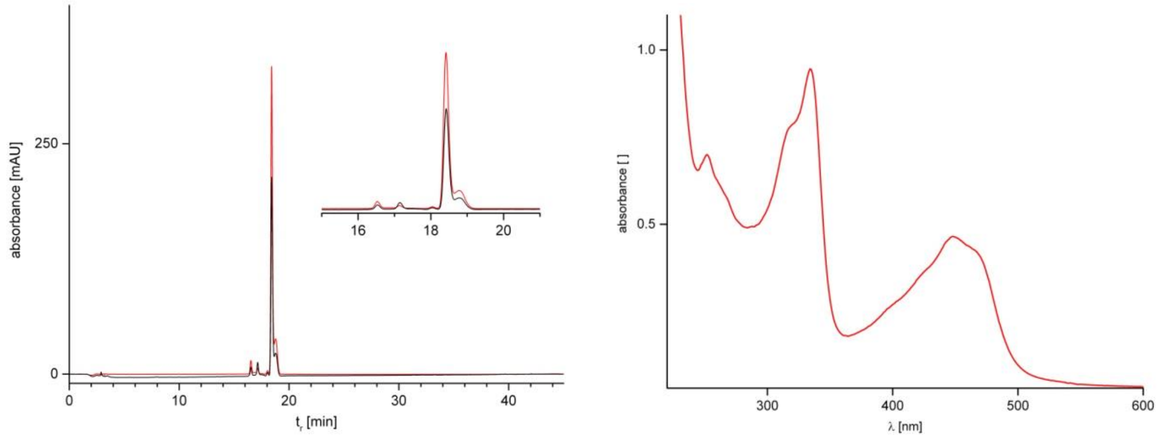

Figure S1. Left: HPLC chromatogram with detection at 280nm (black) and 450 nm (red). Right: UV-Vis spectrum of crystalline **Niby** (c=46 µM) in 10mM aq. Na-phosphate buffer pH 5

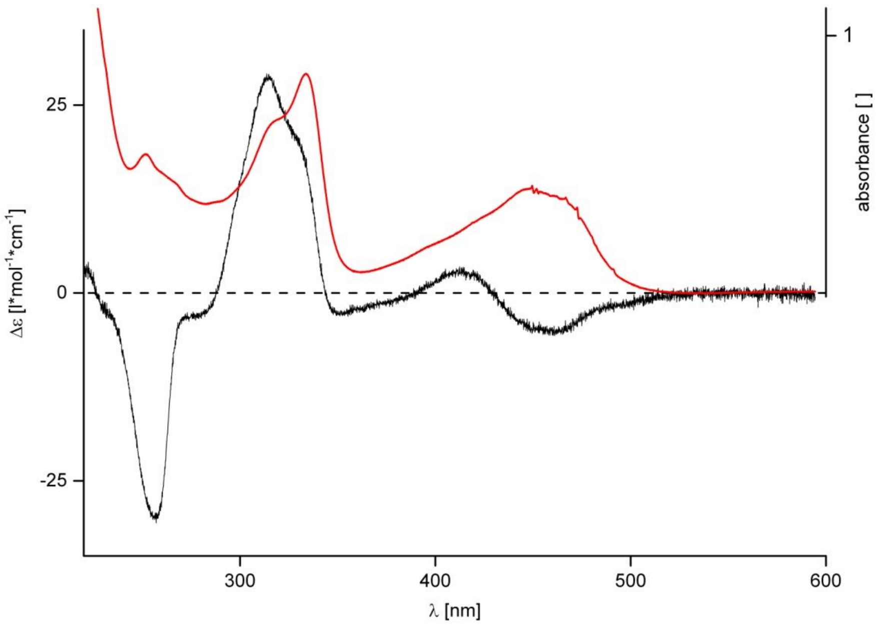

Figure S2: CD-spectrum (black trace) and UV-Vis spectrum (red trace) of crystalline **Niby** (c=46 µM) in 10mM aq. Na-phosphate pH 5 (measured with Jasco J-1500-150 CD spectrometer)

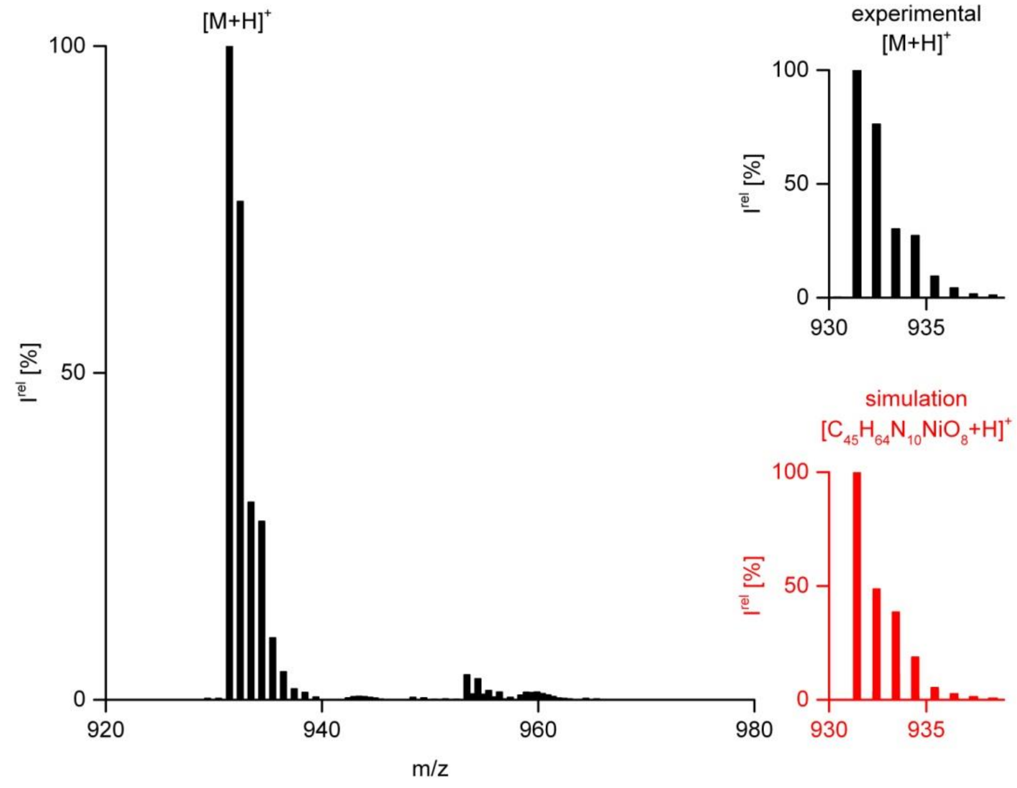

Figure S3: Section of the pseudo-molecular ion $[M+H]^+$ in the HR-ESI mass spectrum of **Niby** (left); comparison of the experimental spectrum of the pseudo-molecular ion $[M+H]^+$ of **Niby** and calculated spectrum for $[C_{45}H_{64}N_{10}NiO_8+H]^+$ (right).

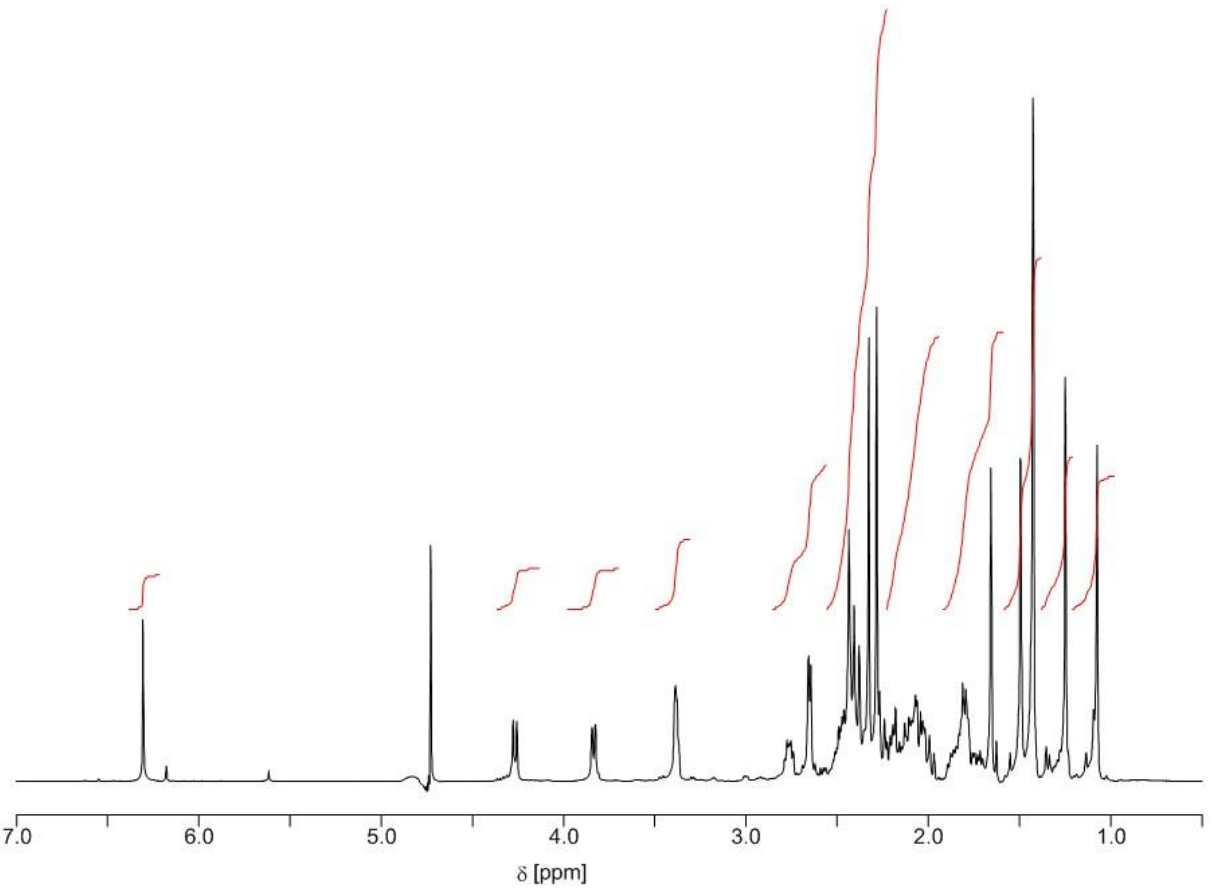

Figure S4: 500MHz ^{1}H-NMR spectrum of crystalline **Niby** (c=1.9 mM) in D_2O, 298 K, HDO-suppression

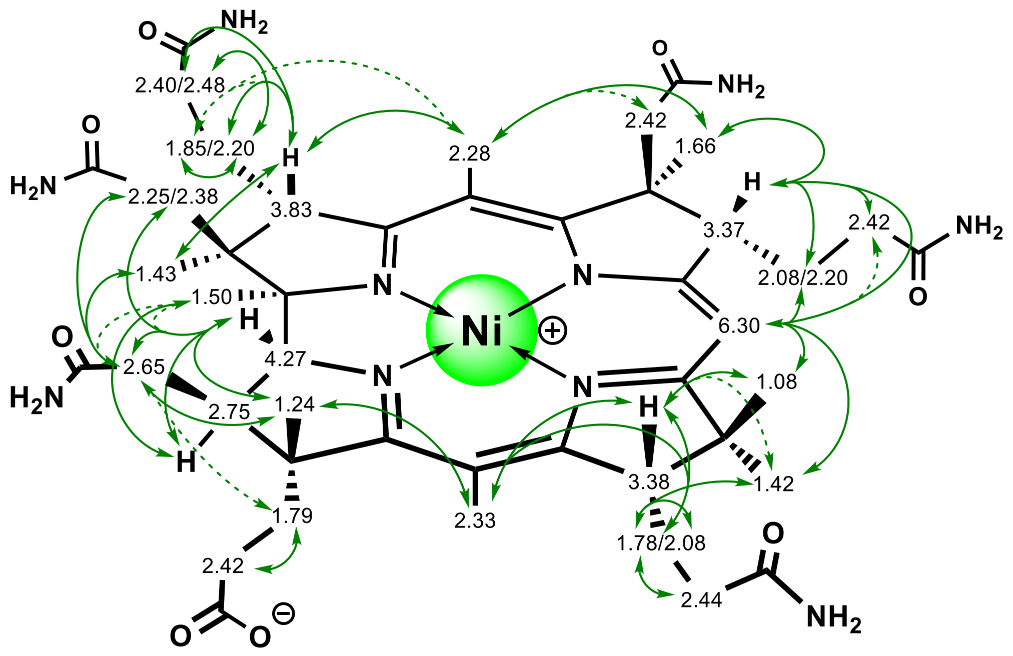

Figure S5: Homonuclear correlations in the 500 MHz ^{1}H,^{1}H-ROESY-spectrum (dashed arrows indicate weak correlations) and derived solution structure of **Niby**

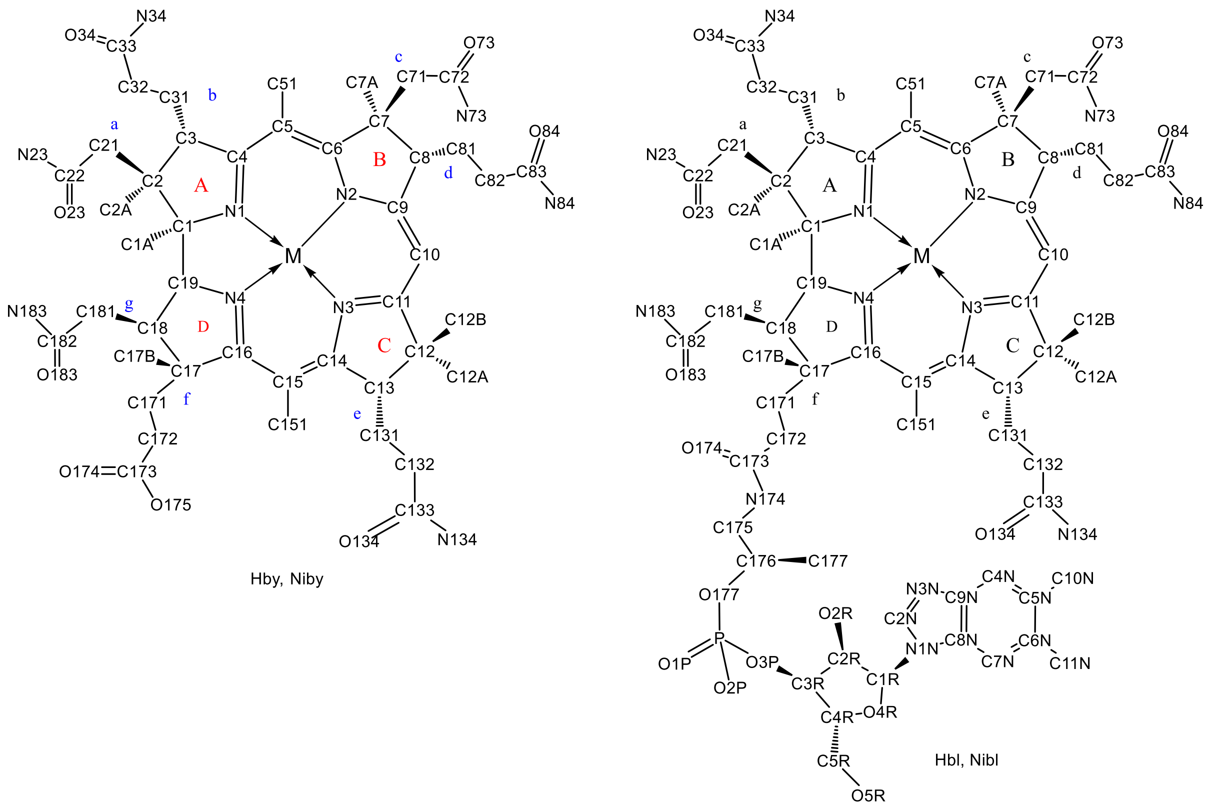

Figure S6: Atom numbering, ring and side chain designations used for **Hby** (M = 2H) and **Niby** (M = Ni, left) and for **Hbl** (M = 2H) and **Nibl** (M = Ni, right).

Table S1: ^{1}H and ^{13}C chemical shifts and signal assignment of **Niby** from 500 MHz NMR spectra in D_2O at 298 K, and chemical shift difference $\Delta\delta=(\delta^{Hby}-\delta^{Nby})$ [ppm] from comparison with spectra of **Hby**[2]; see Figure S6 for atom numbering; * multiplicity not assigned due to signal overlap.

**atom**	**δ ^{13}C [ppm]**	**δ ^{1}H [ppm]**	**multiplicity**	***J* [Hz]**	**Δδ ^{13}C [ppm]**	**Δδ ^{1}H [ppm]**
C1	75.3				5.3	
C1A	23.1	1.50	s		-1.7	-0.26
C2	48.5				-2.2	
C2A	16.6	1.43	s		-1.0	-0.01
C21	42.9	2.25 2.38	AB-system	J_{AB}=10 Hz	-0.4	0.03 -0.03
C22	176.5	-			0.0	
C3	55.1	3.83	d	8.8	0.0	-0.36
C31	25.4	1.85 2.20	m		-1.0	-0.01 -0.12
C32	35.0	2.40 2.48	m		-0.7	-0.02 -0.01
C33	178.3	-			0.1	
C4	172.1	-			6.3	
C5	106.8	-			-1.5	
C51	15.0	2.28	s		-2.0	-0.14
C6	163.2	-			-3.2	
C7	49.9	-			-2.8	
C7A	20.5	1.66	s		-1.9	-0.10
C71	45.7	2.42	*		-1.4	0.15
C72	175.3	-			-0.1	
C8	55.9	3.37	m		-2.5	-0.07
C81	25.4	2.08 2.20	m		-0.8	-0.17 -0.06
C82	31.3	2.42	m		1.0	-0.13
C83	175.4	-			2.8	
C9	170.9	-			-0.7	
C10	94.3	6.30	s		-4.8	-0.68
C11	176.3	-			13.1	
C12	52.8	-			-4.2	
C12A	19.0	1.42	s		-1.0	-0.07
C12B	31.2	1.08	s		-0.8	0.07
C13	52.7	3.38	m		-0.1	-0.20
C131	25.0	1.78 2.08	m		-0.5	0.01 -0.08
C132	35.0	2.44	m		-3.5	-0.11
C133	175.2	-			3.2	
C14	172.4				5.2	
C15	105.6				-2.5	
C151	14.9	2.33	s		-1.8	-0.14
C16	172.5				7.0	
C17	58.3				-0.8	
C17B	18.2	1.24	s		0.7	0.12
C171	32.4	1.79	m		-0.7	0.06
C172	34.9	2.42	m		-2.3	-0.12
C173	176.3	-			4.4	
C18	39.3	2.75	m		0.2	0.17
C181	32.8	2.65	d	5.9	-0.2	0.09
C182						
C19	75.3	4.27	d	10.3	-8.7	0.05

4. Synthesis, isolation, and spectral analysis of hydrogenobalamin (Hbl)

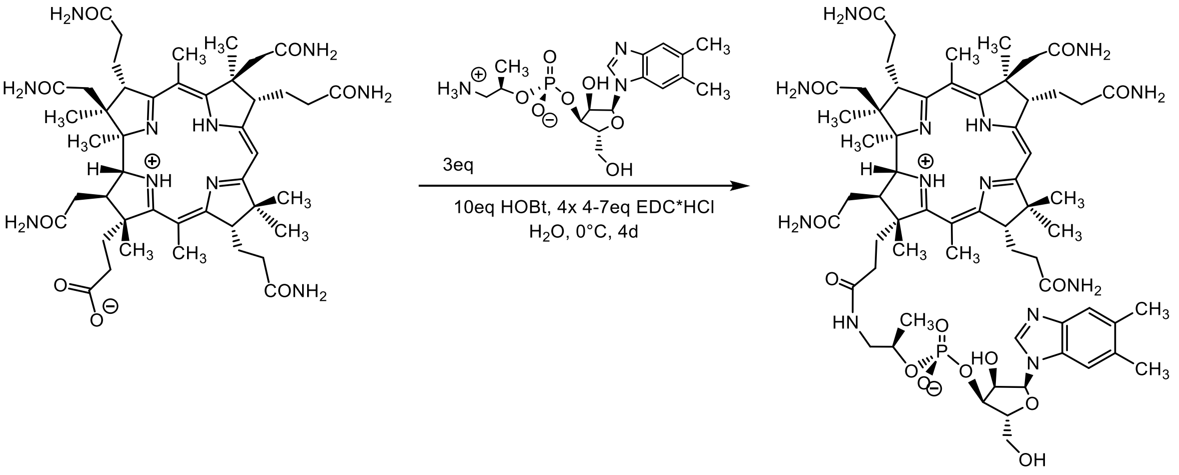

In a 50 mL Schlenk tube 9.12 mg (10.4 µmol) **Hby** [2], 14.71 mg (35.4 µmol, 3.4 eq) B_{12}-nucleotide and 13.2 mg (97.7 µmol, 9.4 eq) 1-hydroxybenzotriazole were dissolved in 11 mL H_2O. The solution was degassed by 3 freeze/vacuum/thaw cycles und overlaid with Ar. 8.85 mg (46.2µmol) EDC*HCl were added to the frozen solution and evacuated (≤ $5*10^{-5}$ bar). The mixture was thawed under Ar and stirred on ice for 5 h. The reaction mixture was kept on ice for 97 h, during which time a total of 40.5 mg (210 µmol, 20.3 eq) of EDC*HCl were added under Ar in three more similar sized portions. The reaction solution was diluted with 10 mL 10 mM NaOAc pH 6 and loaded on a C18-SepPak cartridge. The adsorbate was washed with 20 mL H_2O, 20 mL 100 mM $NaBF_4$ pH 6 and further 20 mL H_2O. The raw red-orange hydrogenobalamin (**Hbl**) was eluted with 6 mL 100 µM $NaBF_4$ in MeOH. The solution was frozen and the solvents were evaporated in HV. The solid crude **Hbl** was dissolved in 10 mL 10 mM NaOAc pH 6 and loaded on the RP18-MPLC column. The crude **Hbl** was purified using 2 L 16%, 1 L 18% and 1 L 20% MeOH in 10 mM NaOAc pH 6. The **Hbl** fraction was collected, frozen in N_2(l) and concentrated in high vacuum to ~100 mL. The concentrate was loaded on a C18-SepPak cartridge and washed with 60 mL H_2O, 40 mL 100 mM $NaBF_4$ pH 6 and further 20 ml H_2O. The **Hbl** was eluted with 3 mL 100 µM $NaBF_4$ in MeOH. The solution was frozen and evaporated in HV. The solid **Hbl** was dissolved in 100µl H_2O and precipitated by the addition of 3 mL MeCN. The mother liquor was removed and the precipitate was dried in high vacuum, yielding 11.3 mg (8.89µmol, 85%) **Hbl** as orange powder.

UV/Vis (c=20.0 µM in 10mM Na-phosphate pH 5, RT): λ^{max} [nm] (lg ε) = 525 (4.11), 499 (4.08), 473 (sh., 3.82), 395 (3.41), 379 (3.47), 330 (4.50), 320 (sh., 4.30), 284 (4.03), 270 (4.37), 239 (3.98)

CD (c=115 µM in 10mM Na-phosphate pH 5, 293K): $\lambda^{max/min}$ [nm] (Δε [$M^{-1}*cm^{-1}$]) =521 (-2.3), 496 (-2.0), 390 (0.3), 327 (8.3), 270 (-5.6), 323 (2.9); λ^0 [nm]= 410, 463, 296, 243

Fluorescence (c=11.0 μM in 10 mM Na-phosphate pH 5, RT):
emission spectrum (excitation at 505 nm): λ^{max}[nm] (I) = 610 (543), 554 (194),
excitation spectrum (emission at 610 nm): λ^{max} [nm] (I) = 526 (657), 505 (546), 392 (95), 376 (111), 329 (805), 305 (sh., 388), 269 (605), 258 (sh., 429)

MS (MeOH): m/z (%) = 1274.661 (7), 1273.658 (24), 1272.655 (33, $[M+H]^+$); 649.326 (6), 648.825 (29), 648.323 (74), 647.822 (100, $[C_{62}H_{90}N_{13}O_{14}P+H+Na]^{2+}$ ≡ $[M+H+Na]^{2+}$), 856.811 (4), 655.809 (21, $[M+H+K]^{2+}$); 637.835 (4), 637.332 (54), 636.831 (76, $[M+2H]^{2+}$);

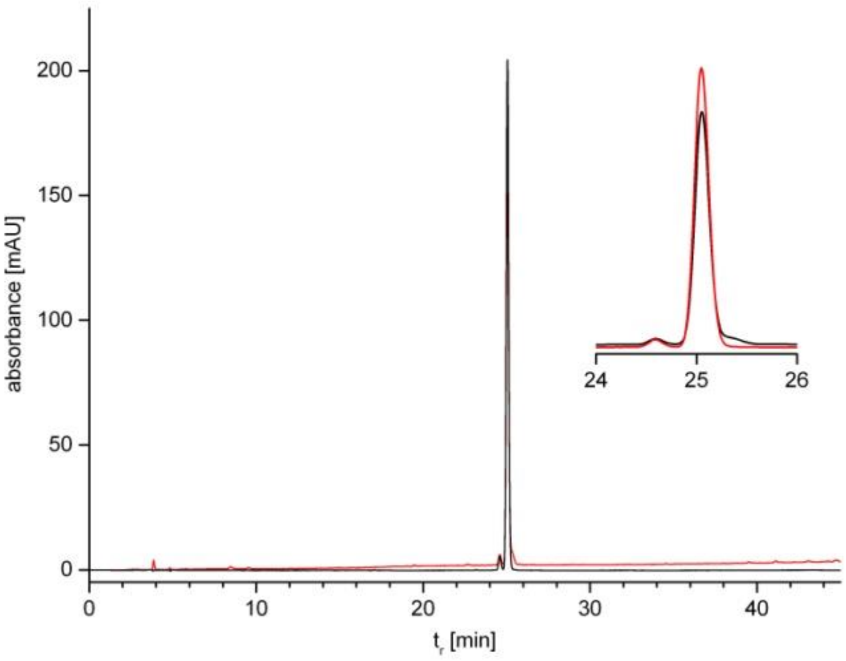

Figure S7: HPLC chromatogram of **HbI** with detection at 280 nm (black) and at 520 nm (red trace).

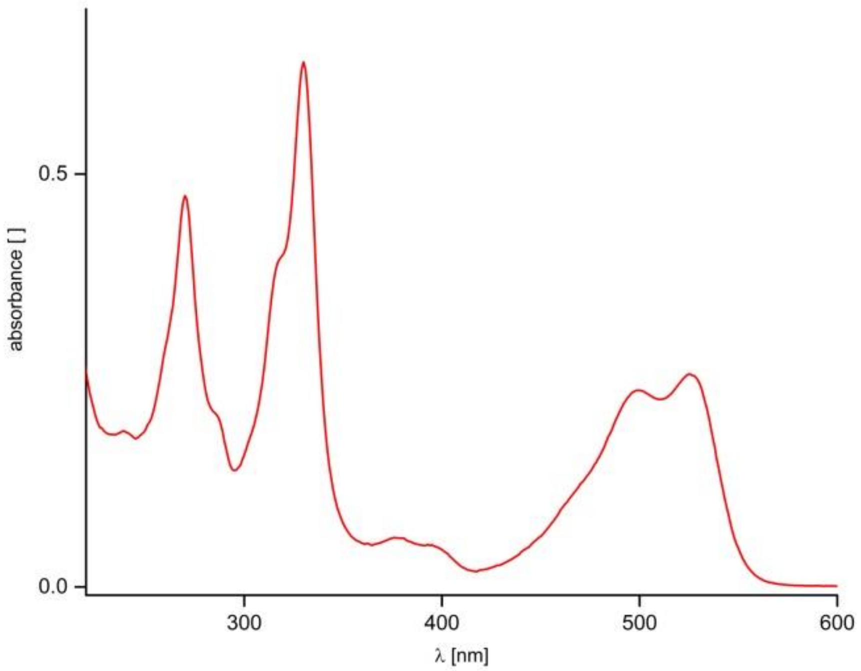

Figure S8: UV-Vis spectrum of **Hbl** (c=20 µM) in 10mM aq. Na-phosphate buffer pH 5.

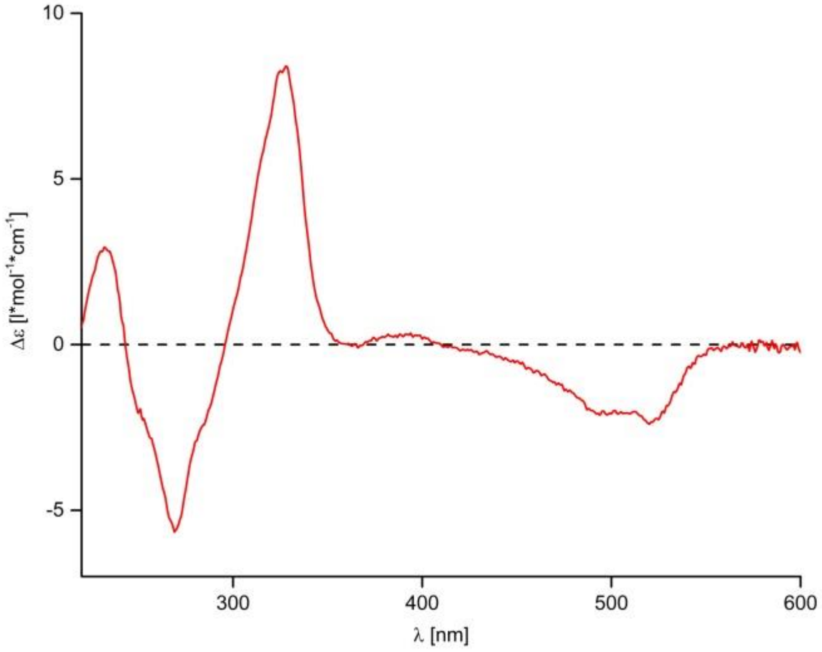

Figure S9: CD spectrum of **Hbl** (c=20 µM) in 10mM aq. Na-phosphate buffer pH 5.

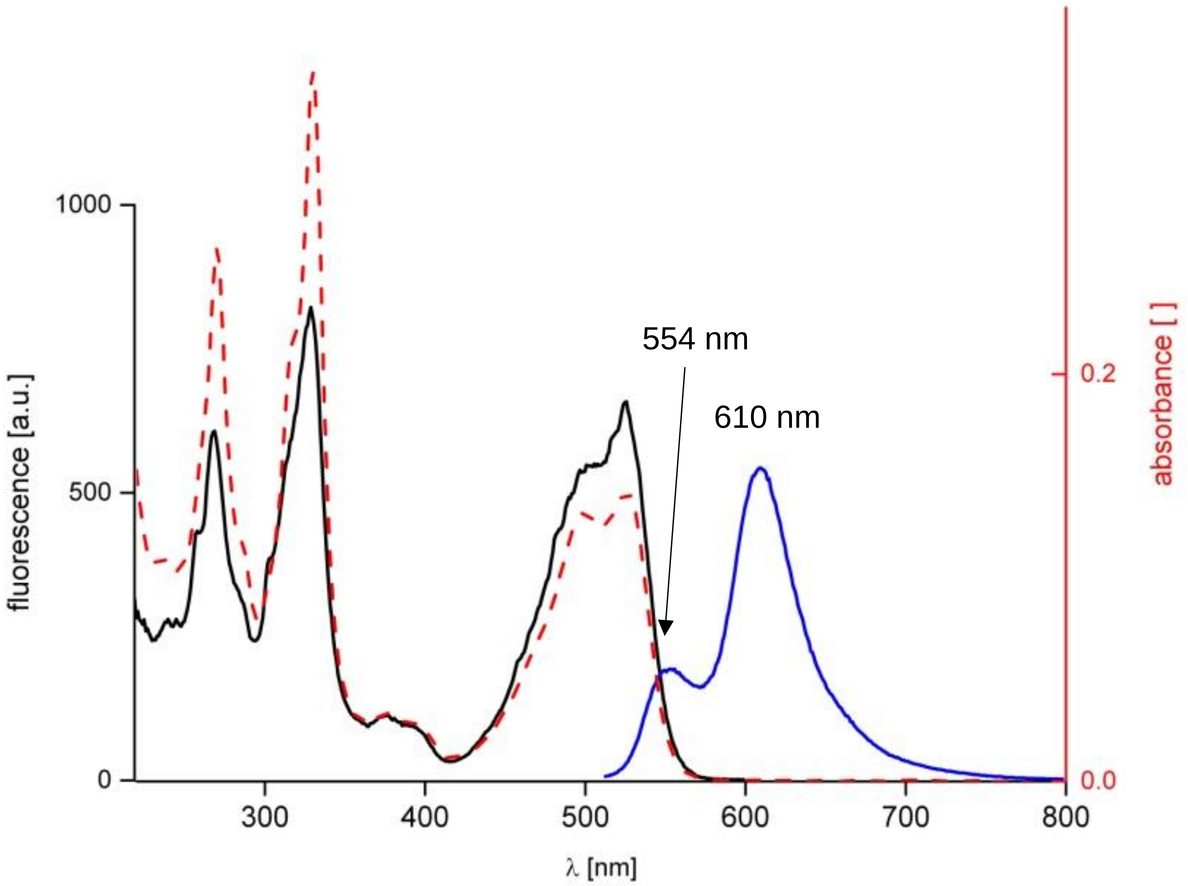

Figure S10: Fluorescence emission spectrum of **Hbl** (blue trace, excitation at 505 nm), fluorescence excitation spectrum (black, emission at 610 nm) and UV-Vis absorption spectrum (dashed red trace) of **Hbl** (c=11 µM in 10mM aq. Na-phosphate pH 5, RT).

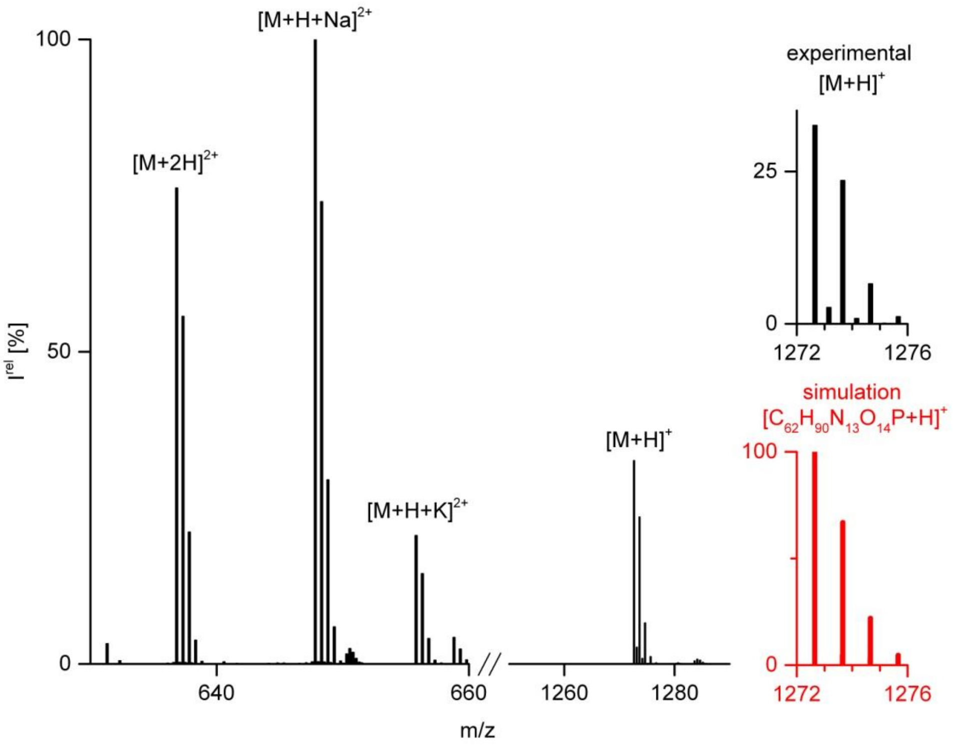

Figure S11: Sections of HR-ESI-MS spectrum of **Hbl** (left); experimental and calculated spectrum for the pseudo-molecular ion $[C_{62}H_{91}N_{13}O_{14}P]^+$ (right).

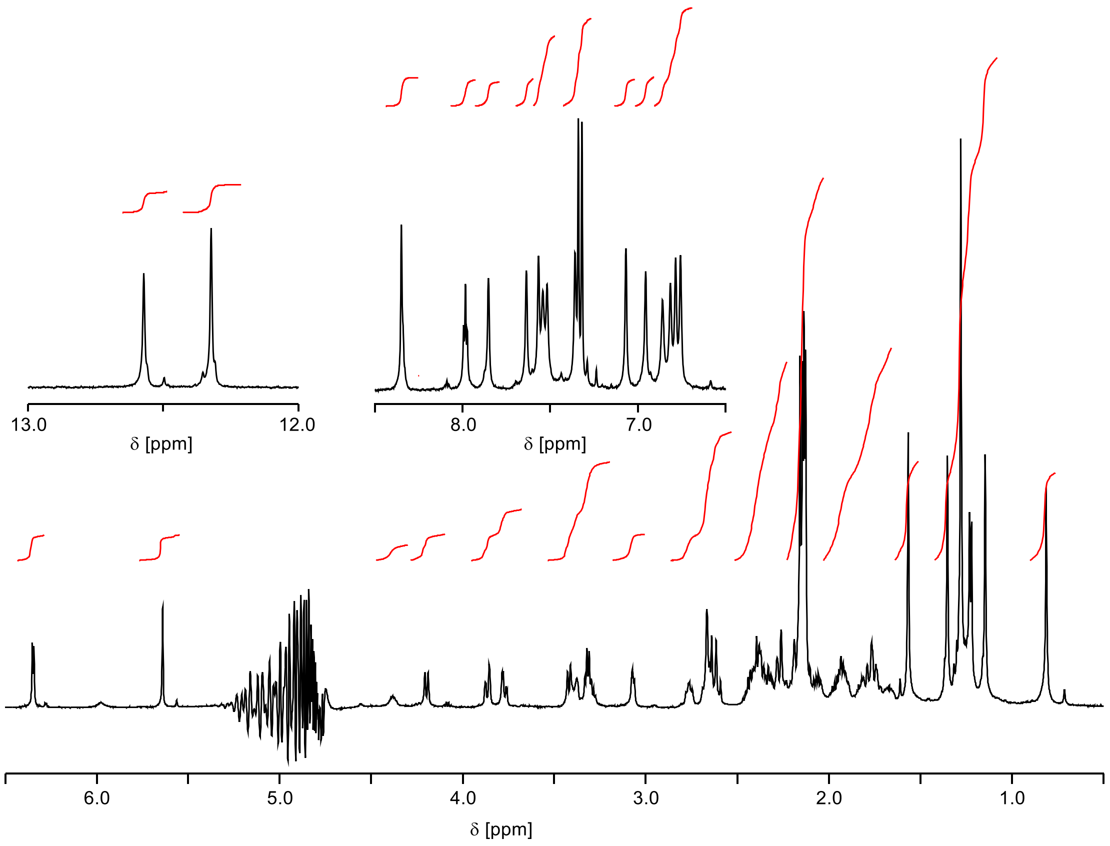

Figure S12: 600MHz ^{1}H-NMR spectrum of **Hbl** (c=1.5 mM in 10 mM aq. Na-phosphate pH 5, H_2O/D_2O (9:1), 298 K), H_2O suppression.

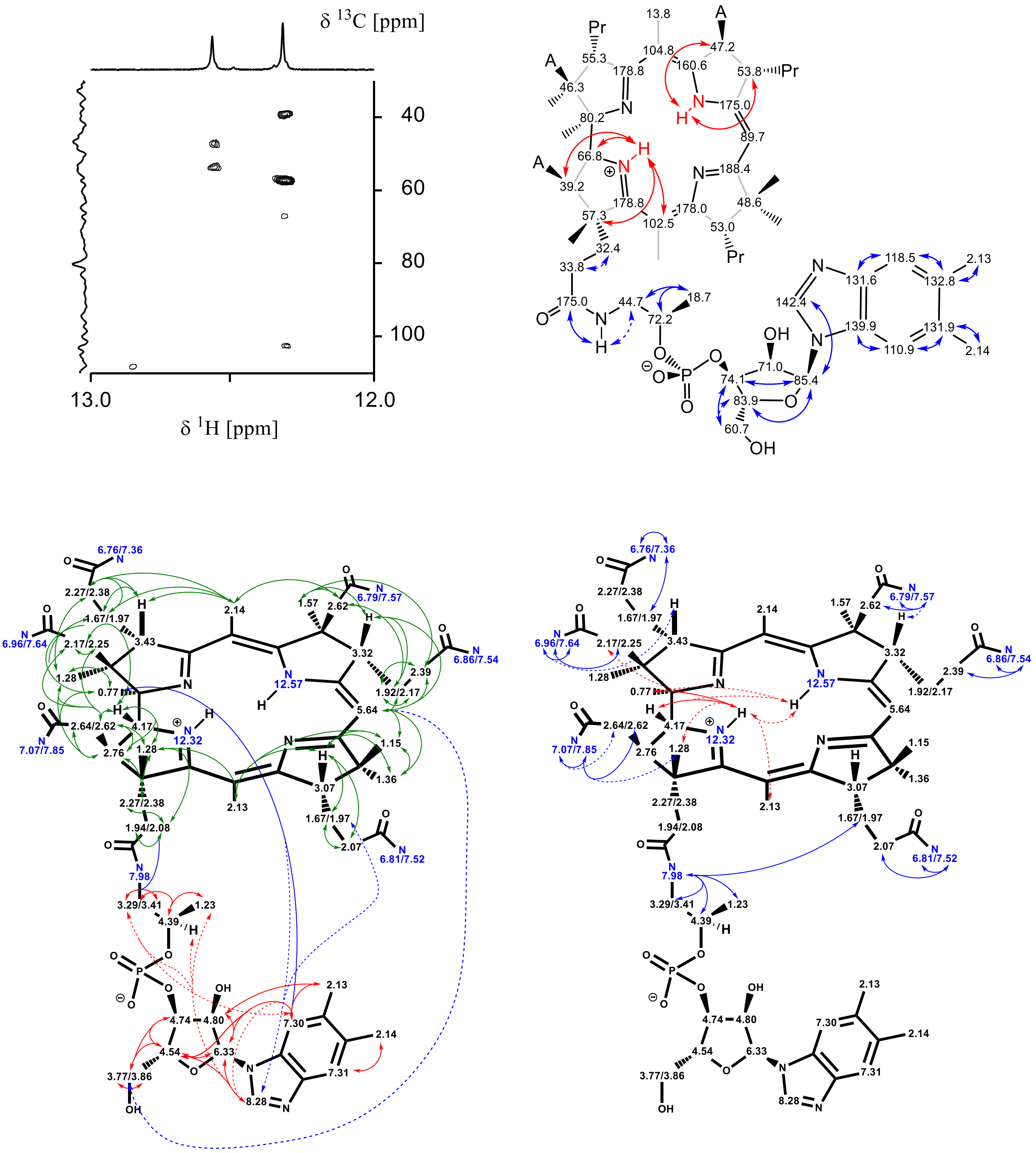

Figure S13: Top, left. Heteronuclear correlations of corrin protons HN2 and HN4 in the low field region of a ^{1}H,^{13}C-HMBC spectrum of **Hbl** in 10 mM aq. Na-phosphate pH 5 H_2O/D_2O (9:1). Top right. Assignments of ^{13}C chemical shift values [ppm] of the corrin and f-side chain and B_{12}-nucleotide carbons and their critical ^{1}H,^{13}C-HMBC correlations. Solution structure, ^{1}H chemical shift values [ppm] and ^{1}H,^{1}H-NOE correlations for carbon-bound protons (left, green arrows), of amide and of the two ‘inner’ corrin protons (right, blue and red arrows, resp.) of **Hbl**; dashed arrows indicate weak correlations.

Table S2a: ^{1}H and ^{13}C chemical shifts and signal assignment of **Hbl** from 600MHz NMR spectra c=1.50 mM in 10 mM Na-phosphate pH 5, H_2O/D_2O (9:1) at 298 K; difference of chemical shifts $\Delta\delta = \delta^{Hby}- \delta^{Hbl}$ [ppm] from comparison with spectra from **Hby**[2], see Figure S6 for atom numbering; * multiplicity not assigned due to signal overlap.

	δ ^{13}C [ppm]	δ ^{1}H [ppm]	multiplicity	*J* [Hz]	Δδ ^{13}C [ppm]	Δδ ^{1}H [ppm]
C1	80.2				0.4	
C1A	21.7	0.77	s		-0.3	0.47
C2	46.3				0.0	
C2A	19.0	1.28	s		-3.4	0.14
C21	42.6	2.16 2.25	AB-system	J_{AB}≈12Hz	-0.1	0.12 0.10
C22	176.2				0.3	
C3	55.3	3.43	m		-0.2	0.04
C31	24.9	1.77 1.83	m		-0.5	0.07 0.25
C32	32.3	2.27 2.38	m		2.0	0.11 0.09
C33	177.8				0.6	
C4	178.2				0.2	
C5	104.8				0.5	
C51	13.8	2.14	s		-0.8	0.00
C6	160.6				-0.6	
C7	47.2				-0.1	
C7A	19.5	1.57	s		-0.9	-0.01
C71	44.5	2.61 2.65	AB-system	J_{AB}≈6Hz	-0.2	-0.04 -0.04
C72	175.1				0.1	
C8	53.8	3.32	m		-0.4	-0.02
C81	25.3	1.92 2.17	m		-0.7	-0.01 -0.03
C82	34.6	2.39	m		-2.3	-0.1
C83	178.0				0.2	
C9	175.0				-4.8	
C10	89.7	5.64	s		-0.2	-0.02
C11	188.4				1.0	
C12	48.6				0.0	
C12A	18.9	1.36	s		-0.9	-0.01
C12B	31.1	1.15	s		-0.7	0.00
C13	53.0	3.07	m		-0.4	0.11
C131	25.4	1.67 1.97	m		-0.9	0.12 0.03
C132	32.2	2.07	m		-0.7	0.26
C133	178.0				0.4	
C14	178.0				-0.4	
C15	102.5				0.6	
C151	13.8	2.13	s		-0.7	0.06
C16	178.8				0.7	
C17	57.3				0.2	
C17B	16.0	1.28	s		2.9	0.08
C171	32.4	2.27 2.38	m		-0.7	-0.42 -0.39
C172	33.8	1.94 2.08	m		-1.2	0.36 0.39
C173	175.0				5.7	
C175	44.7	3.29 3.41	*		0.5	-0.25 -0.23
C176	72.2	4.39	m		-1.9	0.08
C177	18.7	1.23	d	7.0	0.2	0.06
C18	39.2	2.76	dt	8.8/4.4	0.3	0.16
C181	32.7	2.64 2.62	m		-0.1	0.10
C182	178		m		-1.8	
C19	66.8	4.17	d	10.6	-0.2	0.15
C1R	85.4	6.33	d	5.3	1.0	0.11
C2R	71.0	4.74	*		0.6	0.00
C3R	74.1	4.80	*		0.5	0.01
C4R	83.9	4.54	*		0.1	0.05
C5R	60.7	3.77 3.86	ABX	J_A=12.3/3.5Hz J_B=12.3/1.8Hz	0.6	0.05 0.09
C2N	142.4	8.28	s		0.4	0.10
C4N	118.5	7.31	s		0.4	0.24
C5N	132.8				0.3	
C6N	131.9				2.2	
C7N	110.9	7.30	s		0.8	0.16
C8N	139.9				0.2	
C9N	131.6				0.1	
C10N	19.5	2.13	s		0.2	0.25
C11N	19.7	2.14	s		0.3	0.26

Table S4 b: ^{1}H and ^{15}N signal assignment and chemical shift data in 600MHz NMR spectra of **Hbl** (c=1.50 mM in 10 mM Na-phosphate pH 5, H_2O/D_2O (9:1), 298 K); chemical shift differences $\Delta\delta = \delta^{Hby}-\delta^{Hbl}$ [ppm] from comparison with spectra of **Hby**[2], see Figure S6 for atom numbering.

atom	δ^{15}N [ppm]	δ ^{1}H [ppm]		$\Delta\delta$ ^{15}N [ppm]	$\Delta\delta$ ^{1}H [ppm]	
N2	126.2	12.57		-2.0	0.1	
N4	148.0	12.32		-1.8	0.12	
		(Z)	(E)		(Z)	(E)
N23	118.2	6.96	7.64	0.0	0.01	0.01
N34	111.7	6.76	7.36	0.9	0.09	0.17
N73	115.0	6.79	7.57	0.1	-0.01	-0.02
N84	112.5	6.86	7.54	-1.0	-0.11	-0.16
N134	111.6	6.81	7.52	0.0	-0.12	-0.18
N183	114.5	7.07	7.85	-0.3	-0.02	0.04
N174	117.9	7.98 (t, *J*=6.2Hz)		*1.8*	*0.10 (ΔJ=0Hz)*	

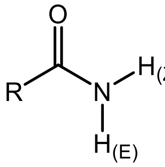

5. Synthesis, isolation, and spectral analysis of nibalamin (Nibl)

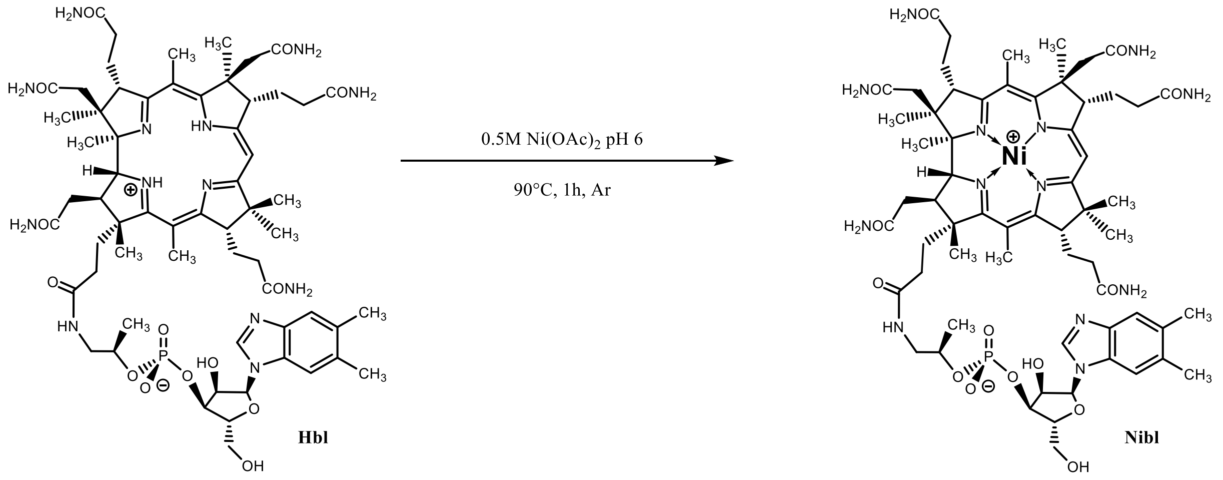

In a 15 mL Schlenk tube equipped with a reflux condenser 0.93 mg (0.73 µmol) **Hbl** were dissolved in 2 mL 0.5 M aqueous $Ni(OAc)_2$ pH 6 and degassed by 7 freeze/vacuum/thaw cycles. The apparatus was pressurized with Ar and heated to 90 °C for 1 h. The reaction mixture was cooled with ice, diluted with 10 mL ice water and loaded on a C18-SepPak cartridge. The adsorbate was washed with 60 ml H_2O, 20 ml 100 mM aq. $NaBF_4$ pH 6 and further 20 mL H_2O. The crude nibalamin (**Nibl**) was eluted with 3 mL 100 µM $NaBF_4$ in MeOH and evaporated on the rotary evaporator (55 °C water bath). The crude **Nibl** was dissolved in 10 mL 10 mM NaOAc pH 6, loaded on the C18-MPLC column and purified with 1 L 20% and 1 L 22% MeCN in aq. NaOAc pH 6. The pure **Nibl** fractions were concentrated to ~5 mL on the rotary evaporator (55 °C water bath), the oily yellow solution was diluted with 10 mM aq. NaOAc pH 6 to 20 mL and loaded on a C18-SepPak cartridge. The adsorbate was washed with 20 mL H_2O, 20 mL 100 µM aq. $NaBF_4$ pH 6 and further 20 mL H_2O. The **Nibl** was eluted with 3 mL 100 µM $NaBF_4$ in MeOH and the methanolic solution was evaporated. The **Nibl** was dissolved in 1 mL H_2O and lyophilized, yielding 0.77 mg (0.56 µmol, 77%) yellow **Nibl**.

UV-Vis (c=57 µM in H_2O): λ^{max} [nm] (lg ε) =465 (sh, 3.86), 448 (3.90), 429 (sh, 3.80), 402 (sh, 3.62), 335 (4.22), 321 (sh, 4.10), 288, (3.94), 279 (3.94), 251 (4.10)

CD (c=57 µM in H_2O, 293K): $\lambda^{max/min}$ [nm] (±Δε [M^{-1}*cm^{-1}]) = 456 (-1.0), 415 (0.6), 352 (-0.6), 329 (sh. 3.9), 315 (5.2), 257 (-5.7); λ^0 [nm] = 429, 393, 343, 290, 229

MS (MeOH): m/z (%)= 1329.578 (6), 1328.575 (8, $[M+H]^+$); 687.271 (7), 683.769 (10, $[M-H+2Na]^{2+}$); 684.271 (6), 683.769 (8, $[M+H+K]^{2+}$); 677.782 (14), 677.282 (41), 676.782 (75), 676.284 (86), 675.782 (100, $[C_{62}H_{88}N_{13}NiO_{14}P+H+Na]^{2+}$ ≡ $[M+H+Na]^{2+}$); 666.291 (8), 665.790 (11), 665.293 (24), 664.791 (30, $[M+2H]^{2+}$);

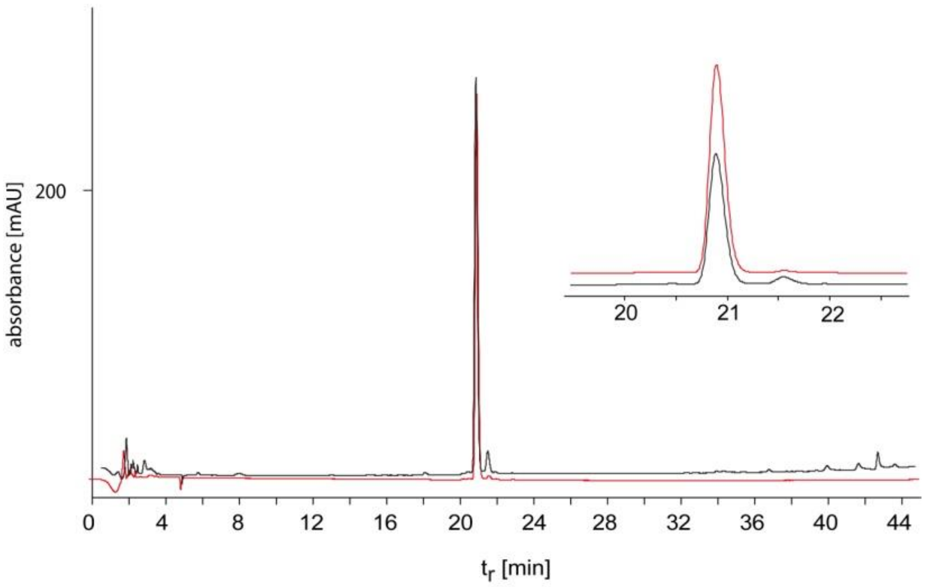

Figure S14: HPLC chromatogram of **NibI** with detection at 280 nm (black trace) and at 520 nm (red trace).

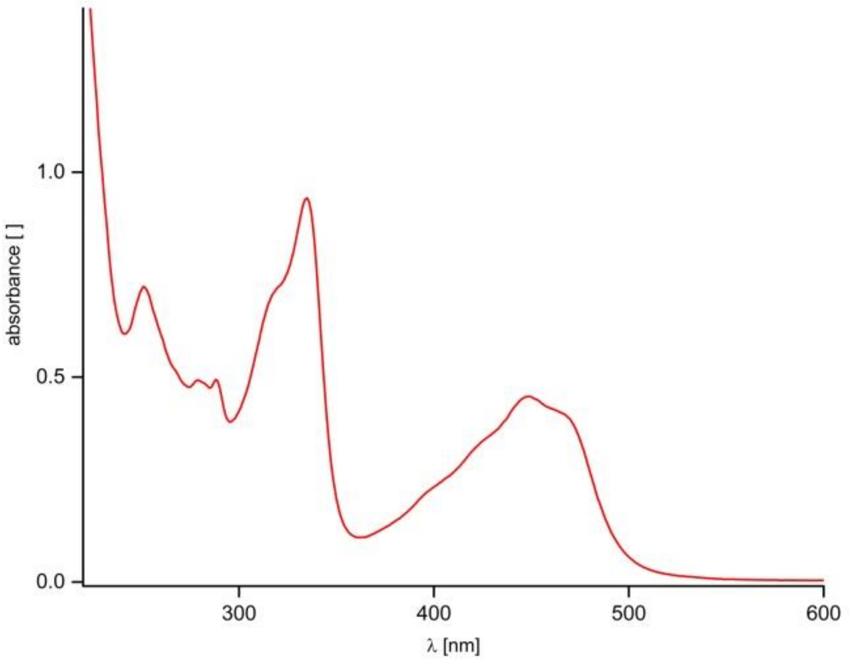

Figure S15: UV-Vis spectrum of **NibI** in H_2O (c=57 µM).

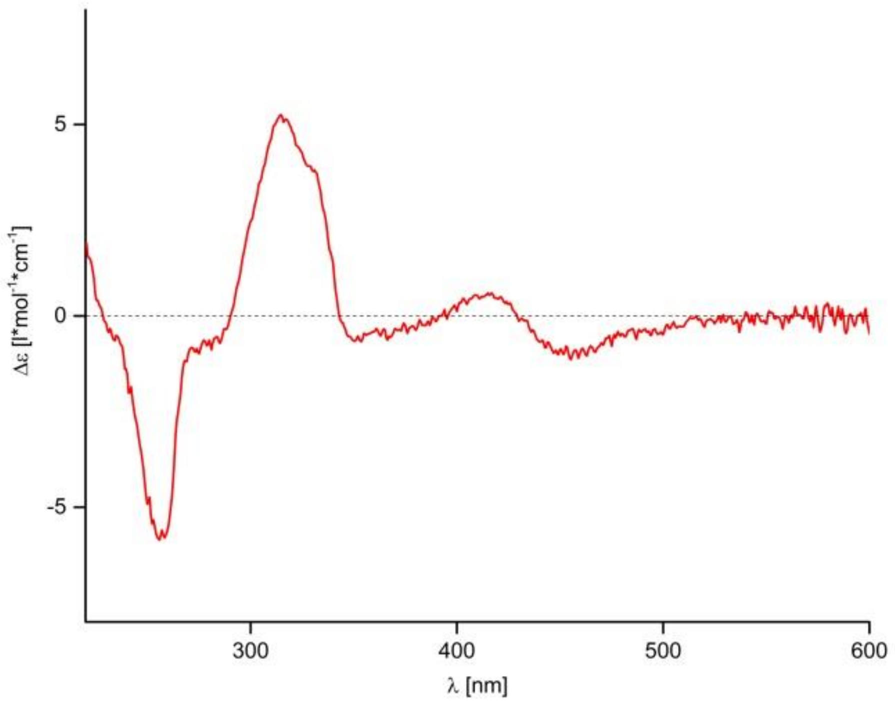

Figure S16: CD spectrum of **NibI** (c = 57 µM) in H_2O.

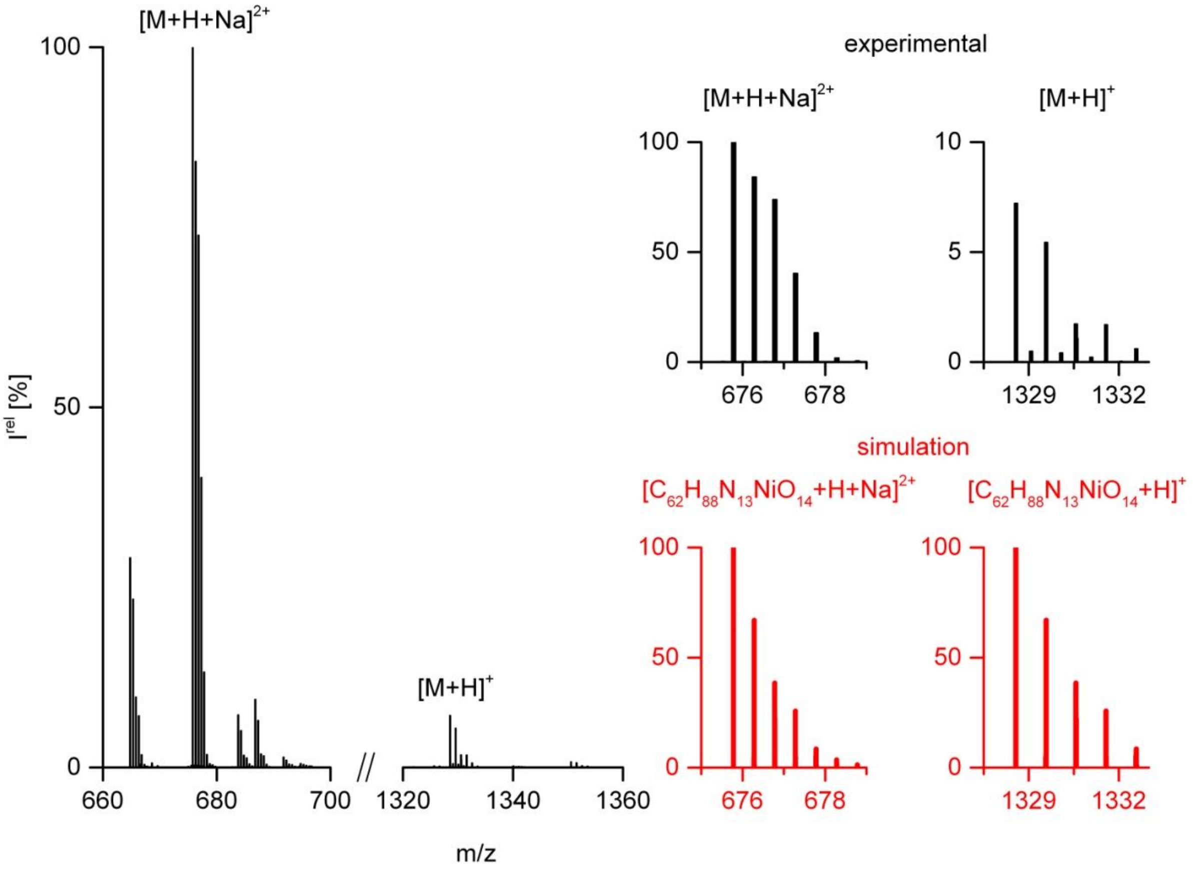

Figure S17: HR-ESI-MS of **NibI** (left); experimental and calculated spectrum for doubly and singly charged pseudo-molecular ions $[C_{62}H_{89}N_{13}NiO_{14}PNa]^{2+}$ and $[C_{62}H_{89}N_{13}NiO_{14}P]^{+}$ (right).

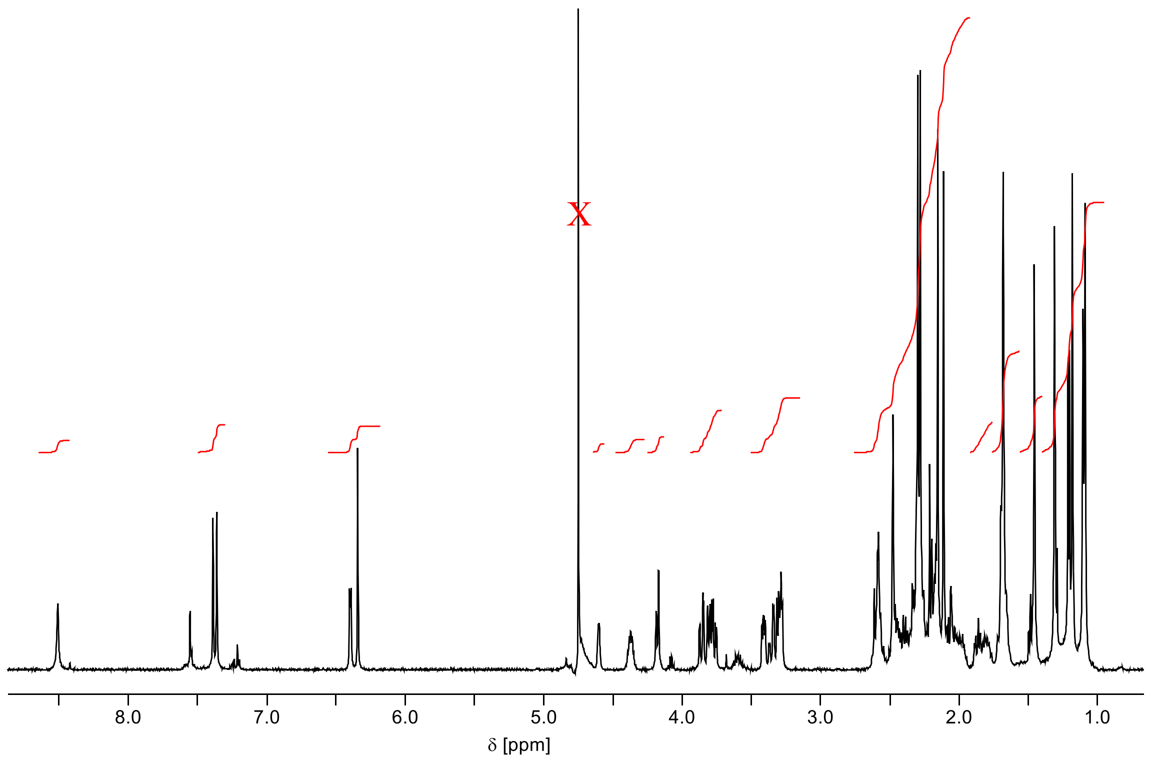

Figure S18: 500 MHz ¹H-NMR spectrum of **NibI** (c=1.4 mM) in D_2O, 298 K, HDO suppression, X= residual solvent signal.

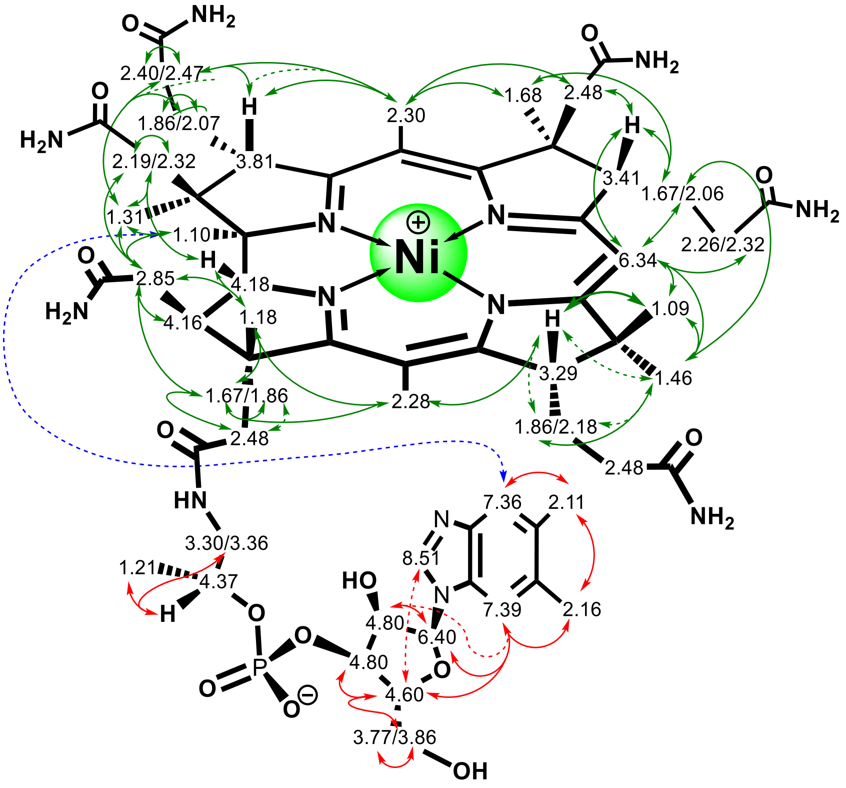

Figure S19: ¹H,¹H-NOE correlations between carbon-bound protons (green and red arrows) of **NibI** in D_2O (298 K) and derived solution structure of **Nibi**; dashed arrows indicate weak correlations.

Table S3: ^{1}H and ^{13}C chemical shifts and signal assignment of **Nibl** from 500 MHz NMR spectra in D_2O at 298 K, difference of chemical shifts $\Delta\delta = \delta^{Hbl} - \delta^{Nibl}$ [ppm] from comparison with spectra from **Hbl**, see Figure S6 for atom numbering,* multiplicity not fully assigned due to signal overlap.

**Atom**	**δ ^{13}C**	**δ ^{1}H [ppm]**	**multiplicity**	***J* [Hz]**	**Δδ ^{13}C [ppm]**	**Δδ ^{1}H [ppm]**
C1	83.7				-3.5	
C1A	22.7	1.10	s		-1.0	-0.33
C2	45.0				1.3	
C2A	16.4	1.31	s		2.6	-0.03
C21	42.8	2.27 2.43	AB-system	J_{AB}≈16	-0.2	-0.11 -0.18
C22	176.0				0.2	
C3	54.7	3.81	d^{app}	8.8	0.6	-0.38
C31	25.3	1.86 2.07	m		-0.4	-0.09 -0.24
C32	34.8	2.40 2.47	m		-2.5	-0.13 -0.09
C33	177.8				0.0	
C4	177.9				0.3	
C5	106.6				-1.8	
C51	14.8	2.30	s		-1.0	-0.16
C6	163.0				-2.4	
C7	49.6				-2.4	
C7A	20.5	1.68	s		-1.0	-0.11
C71	45.9	2.48	s^{app}		-1.4	0.13
C72	175				0.1	
C8	55.6	3.41	dd	8.4/4.1	-1.8	0.09
C81	25.7	1.67 2.06	m		-0.4	0.25 0.11
C82	31.9	2.26 2.32	m		2.7	0.13 0.07
C83	176.0				2.0	
C9	170.5				4.5	
C10	94.3	6.34	s		-4.6	-0.70
C11	175.8				12.6	
C12	46.1				2.5	
C12A	18.9	1.46	s			-0.10
C12B	30.9	1.09	s		0.2	0.06
C13	52.5	3.29	m		0.5	-0.22
C131	31.9	1.86 2.18	m		-6.5	-0.19 -0.21
C132	46.0	2.48			-13.8	-0.41
C133	175				3.0	
C14	165.2				12.8	
C15	105.1				-2.6	
C151	14.6	2.28	s		-0.8	-0.15
C16	171.5				7.3	
C17	57.7				-0.4	
C17B	17.8	1.18	s		-1.8	0.10
C171	31.3	1.67 1.86	m		1.1	0.60 0.52
C172	44.9	2.48	m		-11.2	-0.54
C173	175				0.0	
C175	44.5	3.30 3.36	ABX*	14.7/3.7	0.2	-0.01 0.05
C176	72.4	4.37	m		-0.2	0.02
C177	18.4	1.21	d	5.9	0.3	0.02
C18	54.7	4.16	m		-15.5	-1.40
C181	32.0	2.85	m		0.7	-0.21
C182	176.0				2.0	
C19	74.8	4.18	m		-8.0	0.01
C1R	85.9	6.40	d	4.4	-0.5	-0.07
C2R	74.3	4.80	m		-3.3	-0.06
C3R	71.1	4.80	m		3.0	3.0
C4R	84.7	4.60	q^{app}	2.9	-0.8	-0.06
C5R	61.0	3.77 3.86	dd	12.5/2.9	-0.3	0.00 0.00
C2N	142.4	8.51	s		0.0	-0.23
C4N	111.5	7.36	s		7.0	-0.05
C5N	133.3				-0.5	
C6N	133.7				-1.8	
C7N	117.4	7.39	s		-6.5	-0.09
C8N	137.0				2.9	
C9N	131.1				0.5	
C10N	19.7	2.11	s		-0.2	-0.03
C11N	19.4	2.16	s		0.3	0.03

6. Determination of the pK_a of protonated Nibl

To 50µl of solutions of 100mM K-phosphate puffer pH = 2.04, 2.48, 2.97, 3.98, 4.31, 4.64, 4.95, 5.48, 5.96 and 6.48 containing 1.5 M NaCl, 250 µl H_2O and 200 µl of a **Nibl** stock solution c=98.4 µM inH_2O were added. The UV-Vis spectra of the resulting solutions, containing 39 µM **Nibl** in 10 mM K-phosphate buffer at various pH values and 150 mM NaCl, were recorded at 22±2°C. The pK_a of protonated **Nibl** was calculated from the relative absorption at 251 nm derived from UV-Vis spectra normalized to 261 nm as function of pH.

pK_a (**Nibl-H⁺**) = 4.35 ± 0.06 (in 10mM K-phosphate, 150mM aqueous NaCl)

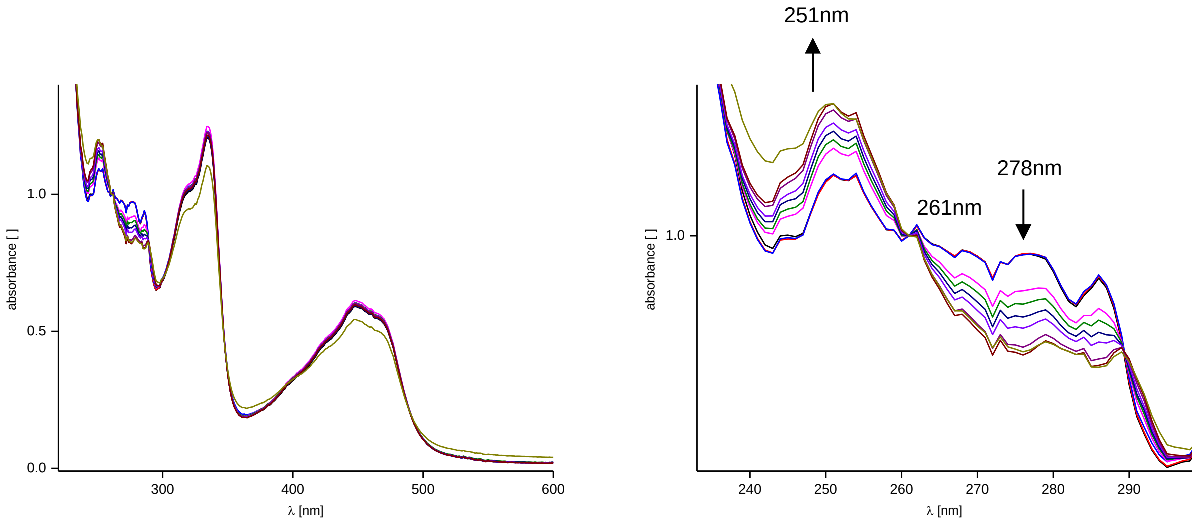

pH	rel. abs^{251nm}	rel. abs^{278nm}
2.04	1.092	0.969
2.48	1.091	0.971
2.97	1.094	0.970
3.98	1.132	0.921
4.31	1.145	0.903
4.66	1.158	0.885
4.95	1.171	0.870
5.48	1.190	0.845
5.96	1.200	0.833
6.48	1.200	0.834

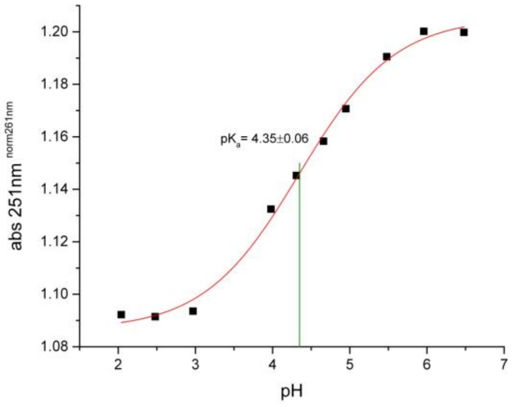

Figure S20: pH-dependence of the UV-Vis spectra of **Nibl** (for the determination of the pKa-value of **Nibl-H⁺** the spectra were normalized to an isosbestic point at 261nm); Bottom right. Plot of the relative absorption at 251nm as function of the pH and pK_a determination at the inflection point of a sigmoidal fitting model yielding a pK_a(**Nibl-H⁺**) = 4.35.

7. X-ray crystal structure analysis of Niby

Crystals of **Niby** were grown from H_2O/MeCN containing ~10 µM $NaBF_4$ at 5±3°C. Data of a small single crystal of **NIby** were collected at 183 K with a Bruker D8 Quest diffractometer (Photon 100 detector) equipped with a microfocus source generator combined with multi-layer optics (monochromatized Mo K_α radiation, λ = 71.073 pm). The structure was solved with SHELXT[8] (version 2014/4) and structure refinement (full-matrix least-squares against F^2) with SHELXL[9] (version 2014/7). Relevant details of the data collection and data evaluation are listed in Table S6.

Supporting crystallographic data of **Niby** may be obtained from the Cambridge Crystallographic Data Centre CCDC deposition service *via* www.ccdc.cam.ac.uk/structures on quoting the deposition number CCDC1971596

Table S4: Crystal data and structure refinement for **Niby.**

empirical formula	$C_{45}H_{64}N_{10}NiO_8$ x 2 H_2O x 2 CH_3CN	
formula weight	1049.91	
temperature	183(2) K	
wavelength	0.71073 Å	
crystal system	monoclinic	
space group	P2₁ (no. 4)	
unit cell dimensions	a = 7.0312(6) Å	a= 90°.
	b = 24.493(2) Å	b= 94.936(2)°.
	c = 15.1975(13) Å	g = 90°.
volume	2607.5(4) $Å^3$	
Z	2	
density (calculated)	1.337 Mg/m3	
absorption coefficient	0.440 mm^{-1}	
F(000)	1120	
crystal size	0.160 x 0.080 x 0.040 mm3	
theta range for data collection	2.139 to 24.998°.	
index ranges	-8<=h<=8, -29<=k<=29, -18<=l<=18	
reflections collected	42185	
independent reflections	9202 [R(int) = 0.0508]	
completeness to theta = 24.998°	99.90%	
absorption correction	Semi-empirical from equivalents	
max. and min. transmission	0.958 and 0.905	
refinement method	Full-matrix least-squares on F2	
data / restraints / parameters	9202 / 5 / 670	
goodness-of-fit on F2	1.04	
final R indices [I>2sigma(I)]	R1 = 0.0391, wR2 = 0.0771	
R indices (all data)	R1 = 0.0530, wR2 = 0.0815	
absolute structure parameter	-0.007(4)	
extinction coefficient	0.0046(6)	
largest diff. peak and hole	0.334 and -0.232 e.$Å^{-3}$	

a

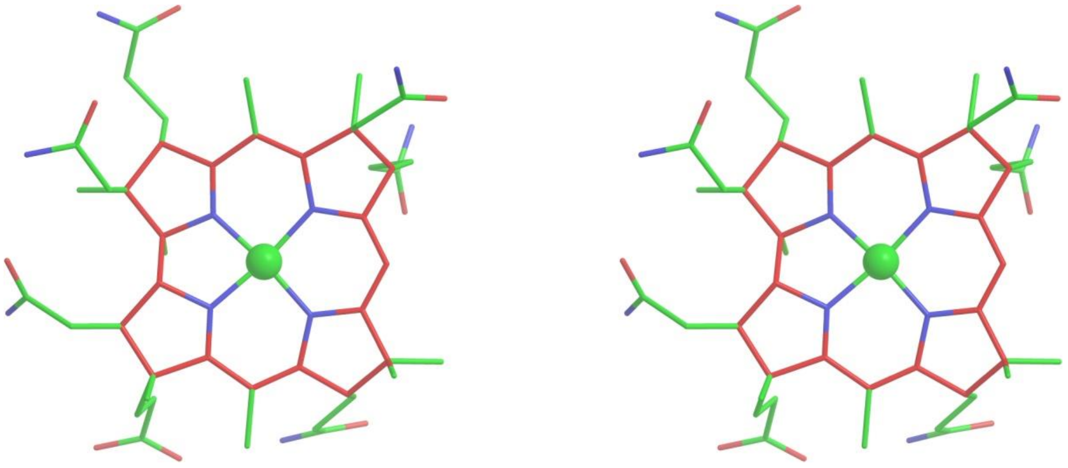

b

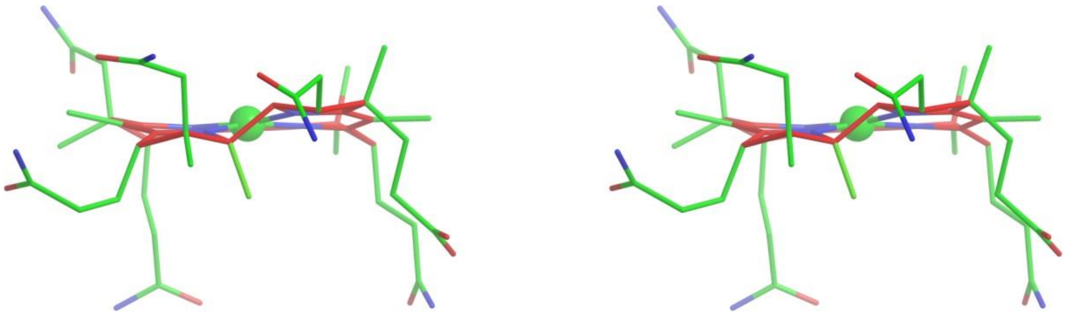

c

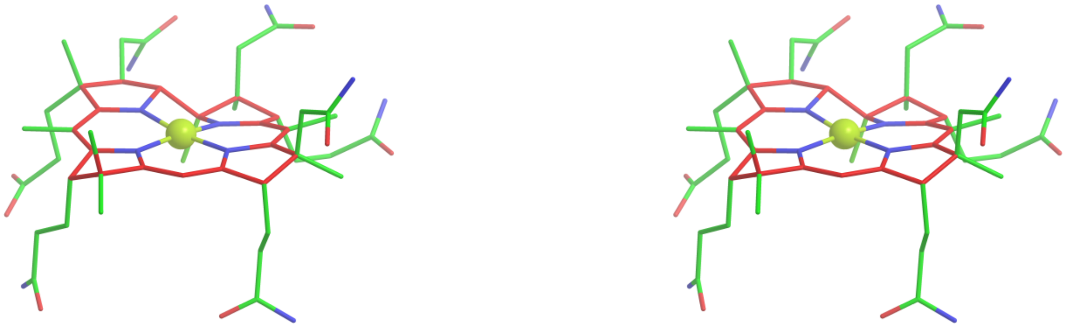

d

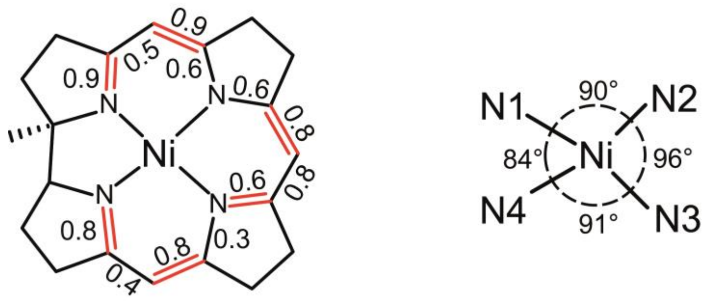

Figure S21: Stereo pictures of the crystal structure of **Niby** (a: top view; b: west-east view; c: west-east view), d left: calculated π-bond orders[3] and d right: experimental Ni-coordination bond angles [°].

Table S5: Crystallographic structure data of **Niby**, **NiCor**[5], **Hby**[2], **Znby**[6] and **Cbin**^{II}[4]. Bond lengths are given in Å dihedral angles in ° (see below for explanations and further illustrations).

	Niby	NiCor [5]	Hby[2]	Znby[6]	Cbin^{II}[4]
d M-N1	1.836	1.851	1.760	2.039	1.889
M-N2	1.882	1.913	2.051	2.031	1.931
M-N3	1.864	1.884	1.923	1.986	1.906
M-N4	1.859	1.847	1.930	2.054	1.874
M-L	-	-	-	2.058/2.095	2.312
N1-N2	2.634	2.689	2.620	2.719	2.676
N2-N3	2.787	2.811	2.996	2.853	2.867
N3-N4	2.654	2.669	2.665	2.770	2.667
N4-N1	2.465	2.449	2.608	2.571	2.538
N1-N3	3.683	3.721	3.668	3.751	3.782
N2-N4	3.730	3.744	3.965	3.937	3.799
M-p(N1-N2-N3-N4) *	+0.025	-0.010	-	+0.624	+0.050
corrin fold angle#	8.71	9.93	11.64	11.64	6.01
corrin helicity *h*§ §§dihedron spanned by N1-N2 -N3-N4[2];	10.09	10.21	12.92	12.92	6.07
Inter-planar angle ϕ $	11.09	10.95	- -	13.15	7.56

*) M-p(N1-N2-N3-N4): distance of the metal center M from the best plane N1-N2-N3-N4

#) corrin fold angle [°]: angle between the best planes A (N1, C4, C5, C6, N2, C9, C10) and B (C10, C11, N3, C14, C15, C16, N4),[7a,b] see Figure S37 for graphical presentations.

§) *h:* corrin helicity [°]: dihedral angle around a virtual dihedron spanned by N1-N2-N3-N4 [2]

$) inter-planar angle ϕ [°]*:* angle between the planes (N1-metal center-N4) and (N2-metal center-N3) [2]

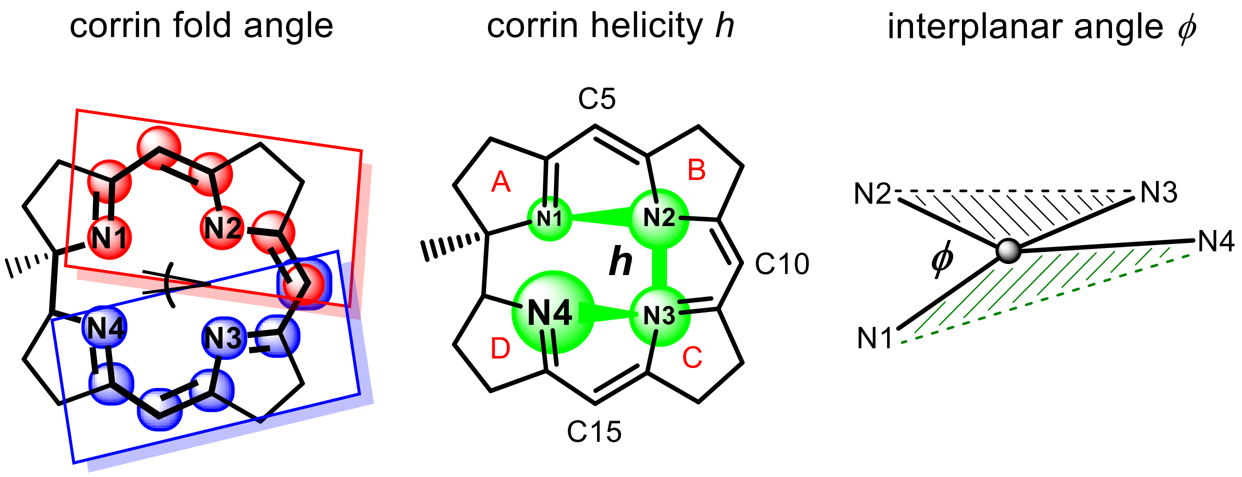

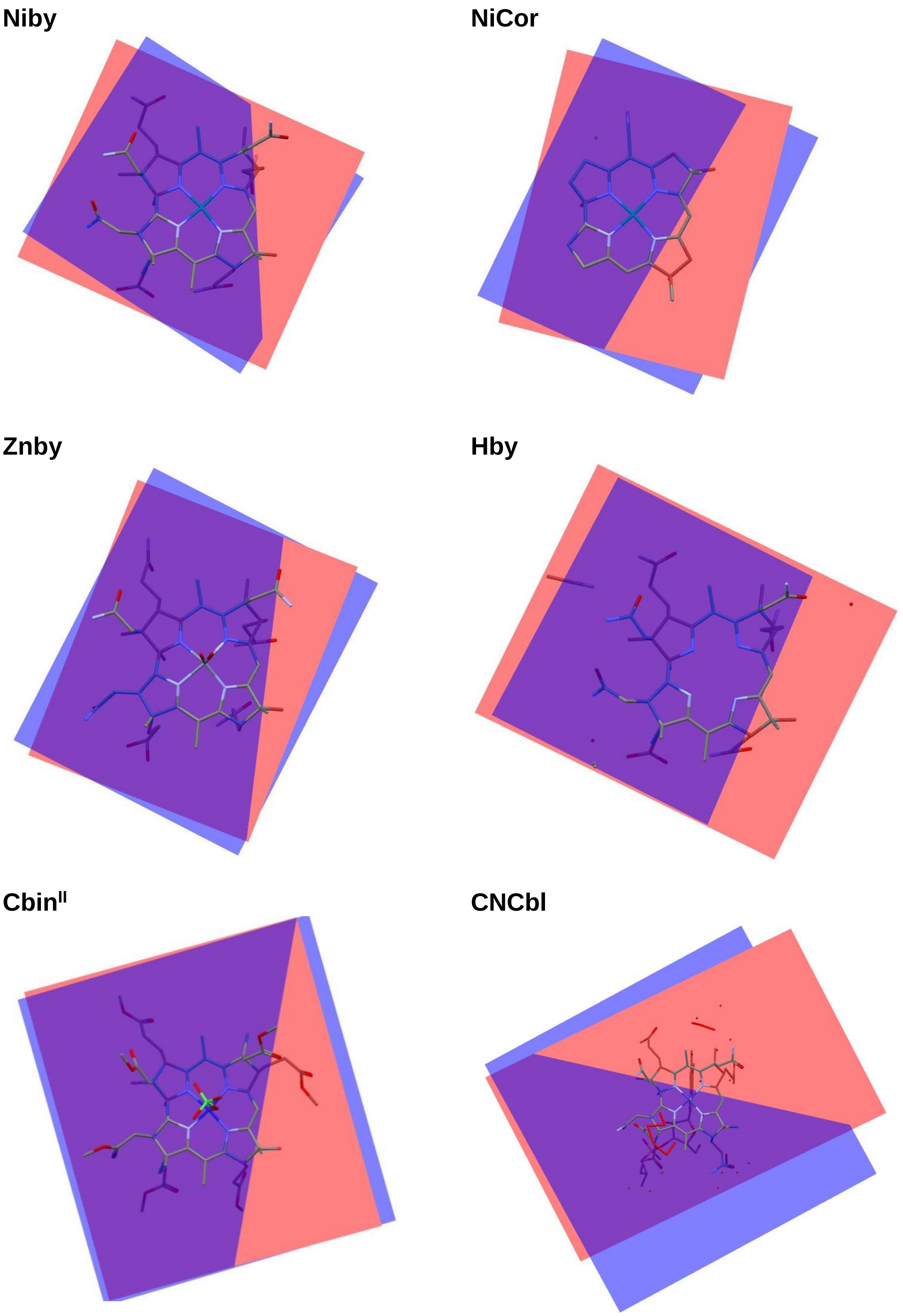

Figure S22: Illustrations of the planes defining the ‘corrin fold angle’, calculated from the corresponding X-ray crystal structures of **Niby**, **NiCor** [5], **Znby** [6], **Hby** [2],**Cbin^{II}** [4] and vitamin B_{12} (**CNCbl**) [7c]. Note the characteristically different basic direction of the intersection between the two planes in the ‘complete’ corrinoid **CNCbl** (bottom, right) and in the structures of the other (all ‘incomplete’) corrins.

8. Computational studies

The X-ray crystal structure of Co(II)-heptamethyl-cobyrinate perchlorate (**Cbin^{II}**) (see Figure S23) was taken as the starting structure for our investigations,[4] as (in previous studies) the amide functions of the natural corrins were prone to form intramolecular H-bonds in the gas phase calculations that are absent in solution and the solid state. In the calculated models, the perchlorate ligand coordinated to the (upper) β-face of the Co(II)-center was substituted by an acetate ion to generate 5-coordinate **Co$_{\beta}$AcO-Cob^{II}in** (one unpaired electron, i.e. a doublet state). In the 5-coordinate diastereoisomer **Co$_{\alpha}$AcO-Cob^{II}in** (one unpaired electron, i.e. a doublet state) the acetate ligand coordinated at the (lower) α-face**.** The axial ligand was omitted from the **Cbin^{II}** structure to give the 4-coordinate **4*c*-Cob^{II}in** (one unpaired electron, i.e. a doublet state). The central Co^{II} ion was changed to Co^{I} generating diamagnetic **Cob^{I}in**, to Ni^{II} to give **Nibin** and (closed shell) Zn^{II} to furnish **4*c*-Znbin**. The structures were subjected to geometry optimizations using Density Functional Theory, where both the BP86[8] and the PBE[9] density functional were tested in combination with the def2-TZVP basis set.[10] Electronic structure calculations on molecules with an odd number of electrons – all Co^{II} structures – were performed as spin unrestricted, alpha and beta electrons were treated with separate spin orbitals, whereas all other calculations were performed as spin restricted. For **Nibin**, both a low spin (diamagnetic) and a high spin (triplet electronic) configuration were calculated but the low spin structure was found to be energetically favored. In order to speed up calculation time the resolution-of-identify (RI) technique was utilized.[11] Dispersion interactions were accounted for by using the empirical D3 corrections with Becke-Johnson damping (BJ).[12] All calculations were performed with Turbomole[13] and structures were visualized with PyMol.[14]

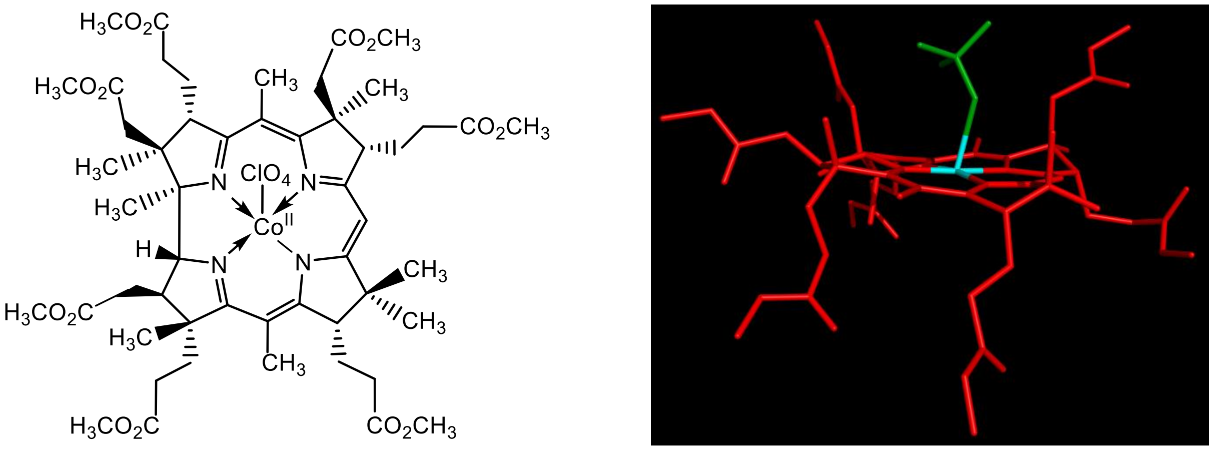

Figure S23. Structural formula of Co(II)-heptamethyl-cobyrinate perchlorate (**Cbin^{II}**) (left) and model of the structure of **Cbin^{II}** with a *5c*-Co(II)-center, coordinated by a perchlorate ligand on its 'upper' β-face (adapted from Figure 7 in [15]).

8.1. Computational studies of 4-coordinate corrin complexes

Table S6: Structural parameters of the optimized structures of the 4-coordinate species **4*c*-Znbin, 4*c*-Cob^{II}in**, **Cob^{I}in**, **Nibin** and of the X-ray crystallographic structure of **Niby.** Bond lengths are given in Å and dihedral angles in °.

	**4*c*-Znbin**		**4*c*-Cob^{II}in**		**Cob^{I}in**		**Nibin**		**Niby**
	BP86/ D3	PBE/ D3	BP86 /D3	PBE/ D3	BP86/ D3	PBE/ D3	BP86 /D3	PBE/ D3	Exp.
N1-N2	2.794	2.813	2.663	2.663	2.639	2.642	2.640	2.642	2.634
N2-N3	2.953	2.967	2.824	2.826	2.802	2.805	2.809	2.812	2.786
N3-N4	2.836	2.822	2.685	2.689	2.651	2.655	2.667	2.672	2.655
N1-N4	2.585	2.587	2.476	2.477	2.451	2.453	2.461	2.463	2.466
N1-N3	3.876	3.911	3.746	3.748	3.707	3.711	3.718	3.722	3.683
N2-N4	3.950	3.935	3.760	3.762	3.724	3.729	3.734	3.738	3.731
M-N1	1.969	1.977	1.857	1.858	1.827	1.829	1.843	1.845	1.836
M-N2	1.974	1.972	1.905	1.906	1.898	1.900	1.893	1.895	1.883
M-N3	1.950	1.957	1.898	1.900	1.888	1.891	1.885	1.888	1.864
N-N4	1.991	1.990	1.865	1.866	1.835	1.837	1.851	1.854	1.859
N1-C4	1.303	1.305	1.311	1.311	1.322	1.322	1.309	1.309	1.299
C4-C5	1.456	1.458	1.432	1.433	1.422	1.424	1.430	1.431	1.431
C5-C6	1.388	1.391	1.378	1.380	1.381	1.382	1.374	1.376	1.354
C6-N2	1.386	1.384	1.398	1.396	1.397	1.395	1.398	1.396	1.409
N2-C9	1.349	1.350	1.359	1.358	1.364	1.363	1.357	1.356	1.352
C9-C10	1.403	1.403	1.386	1.387	1.384	1.385	1.383	1.384	1.378
C10-C11	1.398	1.400	1.384	1.385	1.383	1.383	1.382	1.383	1.367
C11-N3	1.352	1.350	1.360	1.360	1.364	1.363	1.359	1.358	1.352
N3-C14	1.382	1.383	1.392	1.391	1.393	1.391	1.392	1.390	1.408
C14-C15	1.389	1.391	1.380	1.382	1.382	1.383	1.377	1.379	1.364
C15-C16	1.458	1.460	1.434	1.435	1.426	1.427	1.431	1.433	1.438
C16-N4	1.309	1.308	1.317	1.318	1.326	1.327	1.316	1.316	1.304
N4-C19	1.472	1.468	1.488	1.487	1.486	1.484	1.486	1.485	1.494
C19-C1	1.599	1.602	1.553	1.556	1.548	1.551	1.548	1.552	1.538
C1-N1	1.468	1.469	1.490	1.489	1.490	1.489	1.489	1.488	1.492
h	13.24	12.67	6.70	6.94	6.76	7.08	7.59	7.75	10.09
ϕ	16.33	13.90	7.26	7.55	7.28	7.66	8.24	8.42	11.09
$d_{\text{M to plane}}$	+0.089	-0.014	+0.005	+0.009	+0.003	+0.007	+0.007	+0.010	+0.025
$d_{\text{(N1 to plane)}}$	-0.19	-0.18	-0.09	-0.09	-0.09	-0.09	-0.10	-0.10	-0.131
$d_{\text{(N2t o plane)}}$	+0.16	+0.15	+0.08	+0.08	+0.08	+0.08	+0.09	+0.09	+0.116
$d_{\text{(N3 to plane)}}$	-0.16	-0.15	-0.08	-0.08	-0.08	-0.08	-0.09	-0.09	-0.115
$d_{\text{(N4 toplane)}}$	+0.18	+0.18	+0.09	+0.09	+0.09	+0.09	+0.10	+0.10	+0.130

By comparing the structural data of the X-ray crystal structure of **Niby** with those of the computational model **Nibin** derived from **Cbin^{II}** (listed in Table S8), the two structures are deduced to agree closely (see Figure S24). Differences in bond lengths are < 0.04 Å, showing that both density functionals perform very well and that **Nibin** is an excellent model for **Niby**.

To assess the structural similarity of **Nibin** to 4-coordinate Co^{II}, Co^{I}, and Zn^{II} corrin structures that still lack a crystallographic characterization, we calculated computational model structures for these complexes. By keeping the **Cbin^{II}** scaffold as equatorial ligand and only modifying the central metal, its possible axial ligands and its oxidation states, the effect of the metal on the structure of the complex can separately be looked at. As displayed in Table S8, 4-coordinate **4*c*-Cob^{II}in** is structurally very similar to **Nibin**, perhaps only outperformed by **Cob^{I}in**, which shows an even better agreement with **Nibin**. **4*c*-Znbin** on the other hand shows clear differences to **Nibin** and the Co-corrins. In particular, N-N and N-M bond lengths are larger and the Zn center is 0.089 Å on top of the N1-N2-N3-N4 plane, whereas for Nibin and the Co-corrins, the metal lies almost perfectly in this plane.

These computational studies further corroborate that **Nibin** is an excellent structural mimic of Co^{I} and 4-coordinate Co^{II} species.

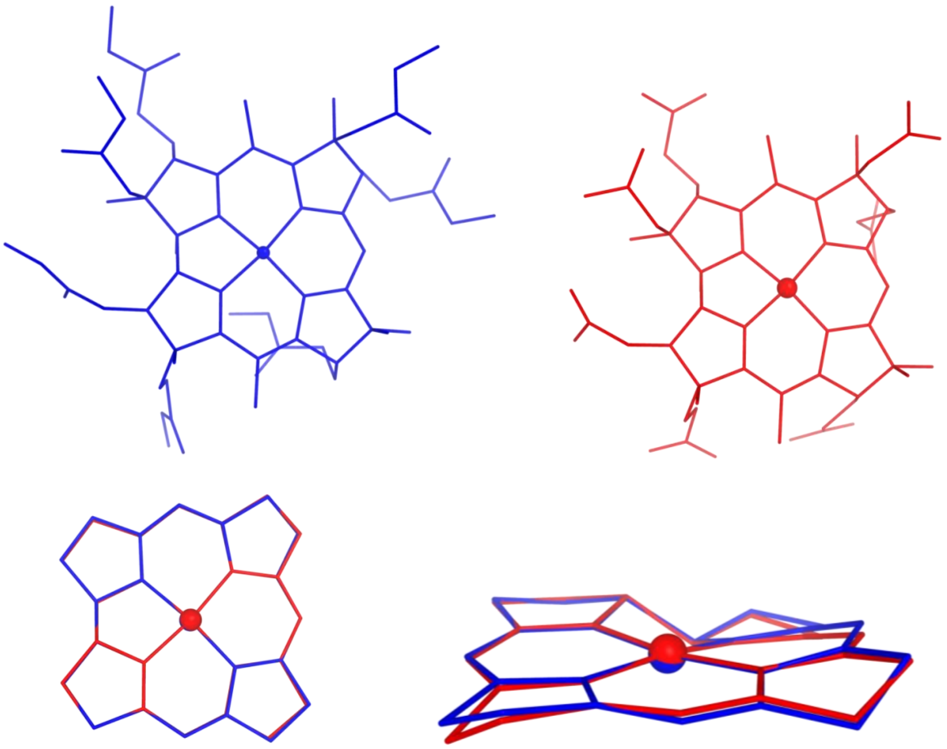

Figure S24: Comparison of the calculated structure of **Nibin** (blue) with the experimental X-ray crystal structure of **Niby** (red), superposition was minimized on the 4 coordinating nitrogen atoms.

8.2. Computational studies with 5-coordinate Co(II)-corrins

Table S7: Structural parameters (in Å and °) of the optimized calculated structures of the 5-coordinate Co(II)-corrins **Co$_β$AcO-Cob^{II}in** and **Co$_α$AcO-Cob^{II}in,** and of the X-ray crystallographic structure of **Cbin^{II}.**

	**Co$_β$AcO-Cob^{II}in**		**Co$_α$AcO-Cob^{II}in**		**Cbin^{II}**
	BP86/D3	PBE/D3	BP86/D3	PBE/D3	Exp.
N1-N2	2.672	2.676	2.665	2.670	2.676
N2-N3	2.805	2.809	2.824	2.826	2.867
N3-N4	2.675	2.679	2.693	2.697	2.667
N1-N4	2.473	2.476	2.463	2.466	2.538
N1-N3	3.740	3.745	3.758	3.764	3.782
N2-N4	3.757	3.761	3.752	3.757	3.799
M-N1	1.860	1.862	1.852	1.854	1.889
M-N2	1.908	1.911	1.905	1.908	1.931
M-N3	1.900	1.904	1.912	1.916	1.906
N-N4	1.855	1.857	1.866	1.868	1.874
N1-C4	1.310	1.311	1.308	1.309	1.289
C4-C5	1.430	1.432	1.429	1.431	1.464
C5-C6	1.379	1.381	1.375	1.377	1.358
C6-N2	1.390	1.389	1.390	1.389	1.414
N2-C9	1.355	1.355	1.353	1.352	1.369
C9-C10	1.384	1.385	1.387	1.388	1.368
C10-C11	1.379	1.381	1.383	1.384	1.397
C11-N3	1.361	1.360	1.359	1.358	1.361
N3-C14	1.387	1.385	1.383	1.381	1.431
C14-C15	1.378	1.380	1.383	1.385	1.397
C15-C16	1.432	1.433	1.432	1.433	1.451
C16-N4	1.312	1.313	1.317	1.318	1.295
N4-C19	1.477	1.476	1.485	1.484	1.522
C19-C1	1.555	1.558	1.552	1.556	1.559
C1-N1	1.487	1.486	1.489	1.489	1.505
h	-5.469	-5.420	-4.827	-4.845	-6.066
ϕ	11.1	11.3	9.65	10.11	7.584
d $_{\text{M to plane}}$	0.110	0.112	-0.095	-0.101	0.047
d$_{\text{(N1 to plane)}}$	-0.072	-0.071	-0.064	-0.064	-0.079
d$_{\text{(N2t o plane)}}$	0.063	0.063	0.056	0.056	0.070
d$_{\text{(N3 to plane)}}$	-0.063	-0.062	-0.055	-0.056	-0.070
d$_{\text{(N4 toplane)}}$	0.072	0.071	0.064	0.064	0.079

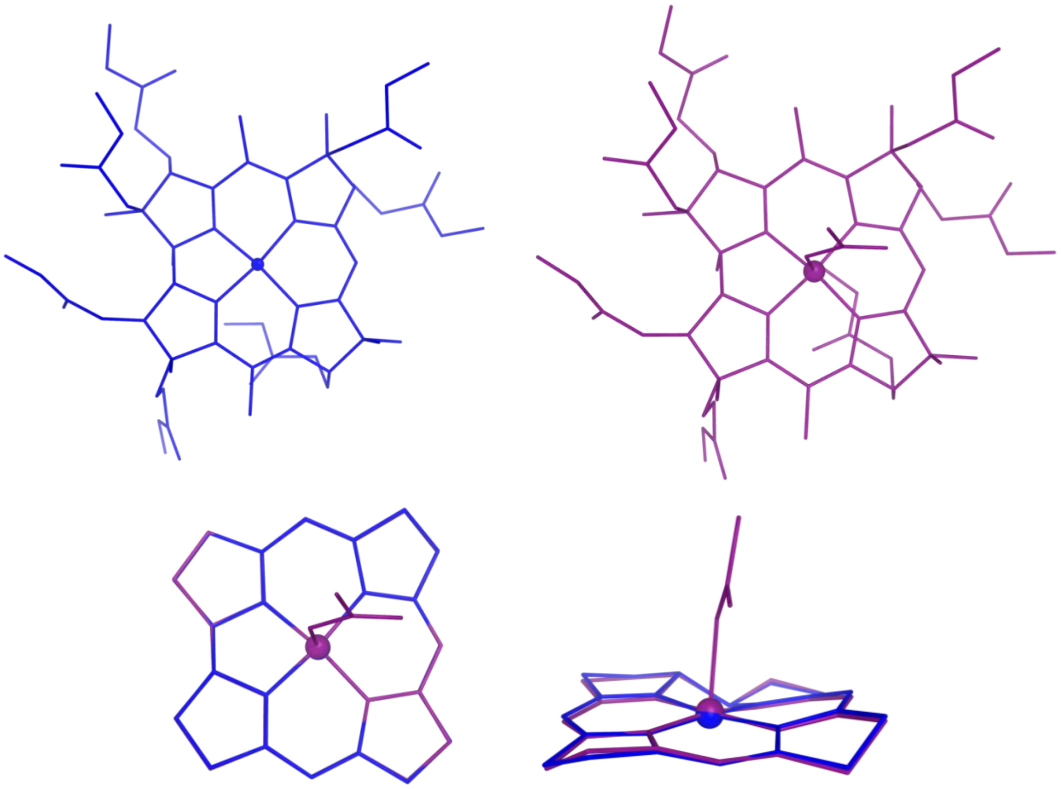

Figure S25: Comparison of the calculated structures of **Nibin** (blue) and of **Co$_{\beta}$AcO-Cob^{II}in** (purple); the superposition was minimized on the 4 coordinating nitrogen atoms.

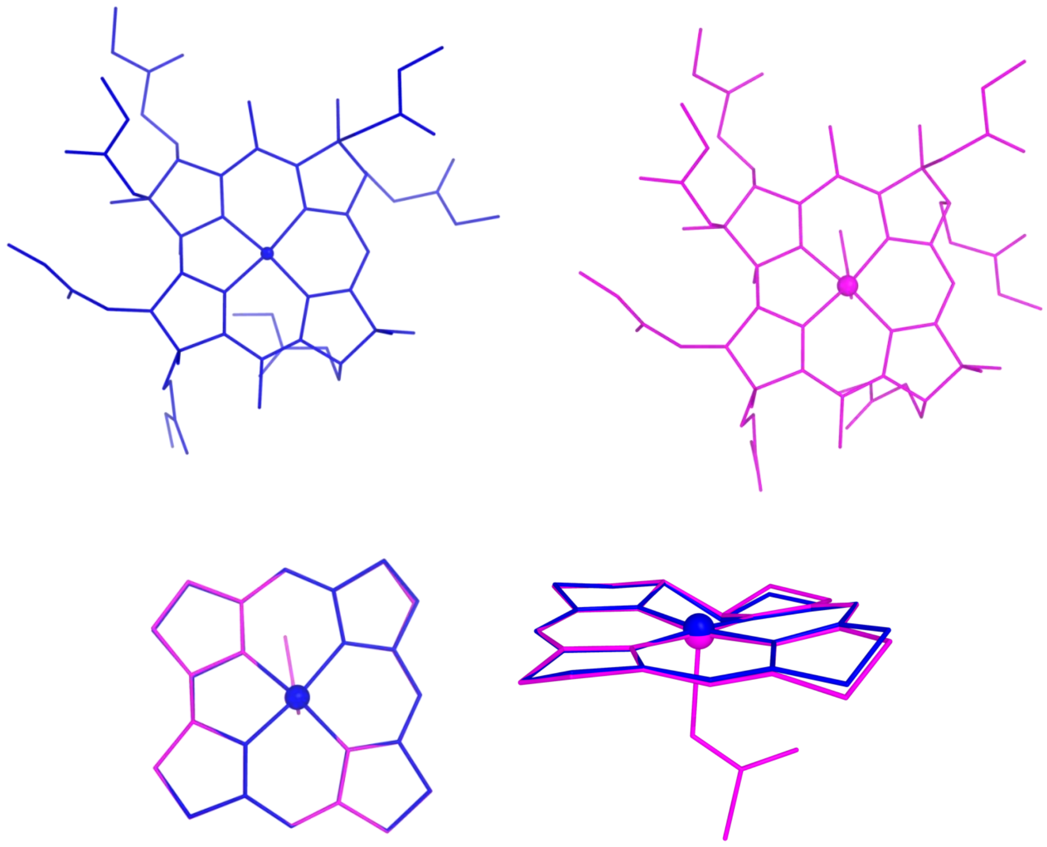

Figure S26: Comparison of the calculated structures of **Nibin** (blue) and of ***5c*-Co$_{\alpha}$AcO-Cob^{II}in** (magenta); the superposition was minimized on the 4 coordinating nitrogen atoms.

9. Inhibition studies with NibI and adenosyltransferase

Adenosyltransferase assays were peformed as previously reported [16] using a recombinant *Brucella melitensis* adenosyltransferase (BtuR). Reactions were undertaken and spectra recorded in a glove box under an atmosphere of nitrogen with less than 5 ppm oxygen. The *B. melitensis* BtuR was purified on a nickel-sepharose column after being recombinantly produced in *E. coli* as previously described [17]. Assays were performed in a 1 mL cuvette containing Tris-HCl buffer, pH 8.0, with 0.4 mM ATP, 0.8 mM $MnCl_2$, 0.75 µM BtuR and varying amounts of **NibI** (1 µM and 5 µM). The reaction was initiated by the addition of 30 µM Co(I)cobalamin, which was prepared from hydroxocobalamin by the addition of 100 µM sodium borohydride. The spectrum of hydroxocobalamin before and after reduction is shown in Fig S27. The effect of reductant on **NibI** was also checked where, apart from a slight dilution effect, the addition of 100 µM borohydride had no effect upon the spectrum (Fig S28). The assay was performed at 37 ˚C and the reaction monitored at 525 nm for up to 5 mins. Control experiments missing just the enzyme, BtuR, showed no activity, whilst the addition of 1 µM or 5 µM **NibI** were found to increasingly inhibit the enzyme activity (Fig S29).

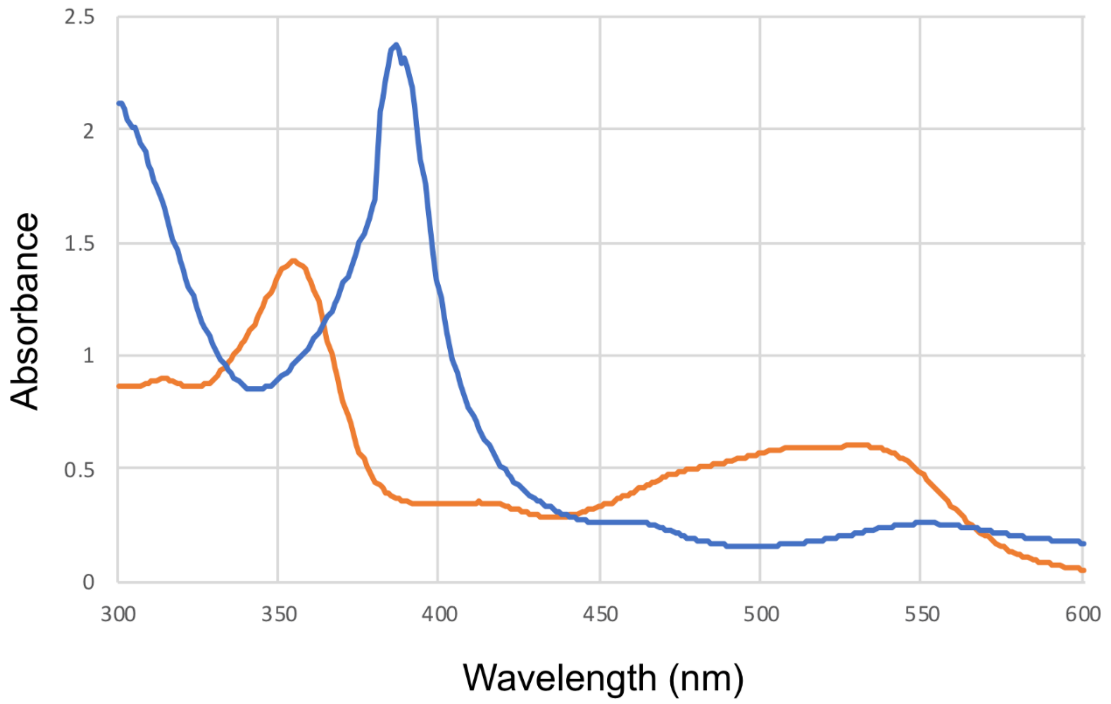

Figure 27. Hydroxocobalamin in the presence and absence of reductant. Spectrum of hydroxocobalamin is shown in orange and after addition of 100 µM sodium borohydride (blue line).

Figure S28

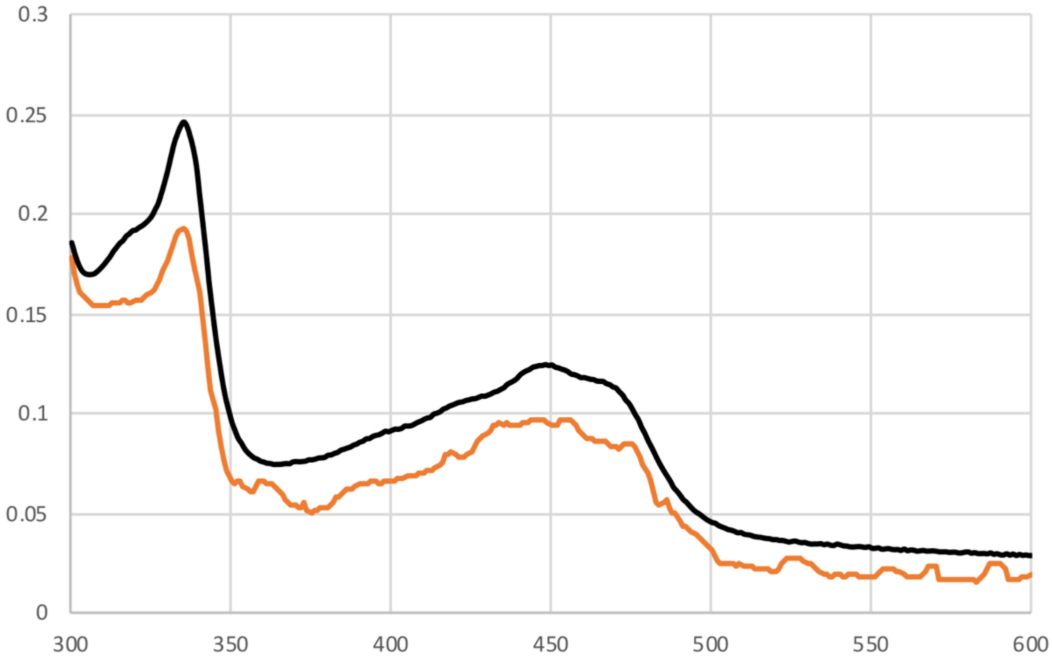

Figure S28. Addition of reductant to Nibl. Spectrum of Nibl before (black) and after (orange) addition of 100 µM sodium borohydride. Slight decrease in absorption is attributable to a small dilution associated with addition of the reductant.

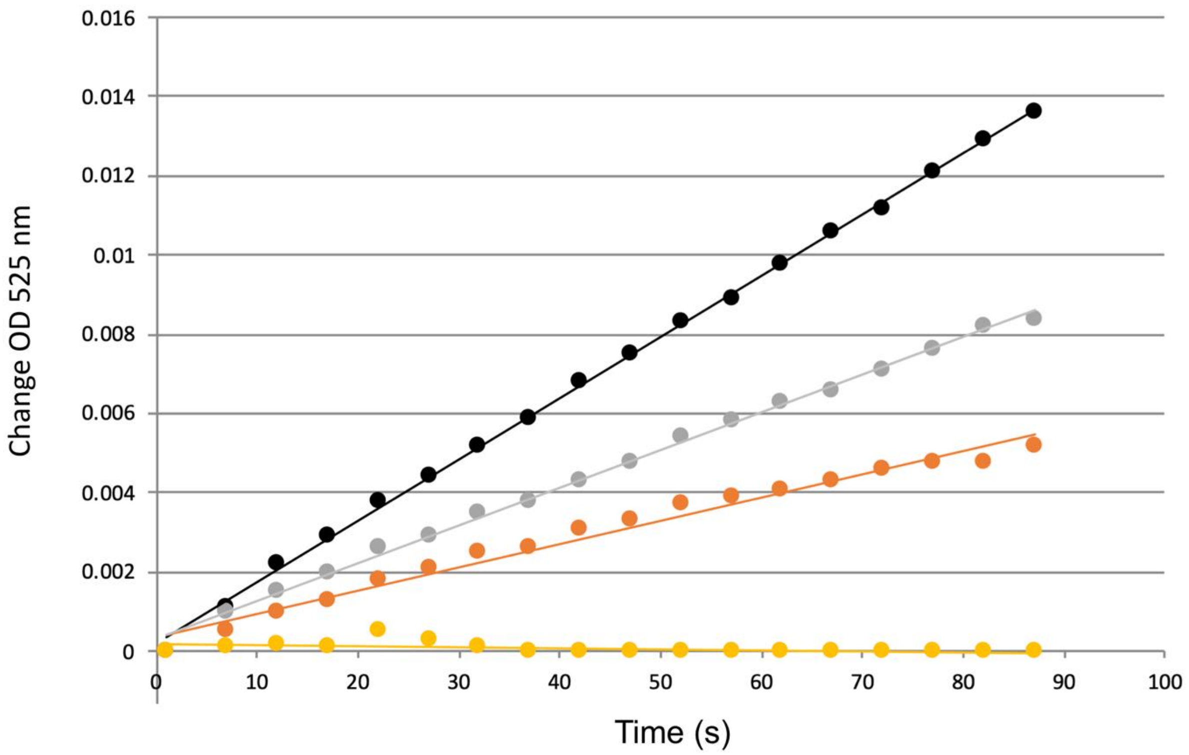

Figure S29. Adenosyltransferase assays conducted with the *B. melitensis* BtuR and different concentrations of **Nibl**. The reactions were monitored at 525 nm. The rate of reaction in the absence of **Nibl** is shown in black, with 1 µM **Nibl** in grey and with 5 µM **Nibl** in orange. A control reading missing the enzyme is shown in yellow. The presence of 1 µM **Nibl** resulted in a 38% reduction and 5 µM **Nibl** a 60% reduction in the activity of the enzyme, demonstrating that **Nibl** acts as a strong inhibitor.

10. References

[1] a. A. Eschenmoser, *Angewandte Chemie International Edition in English* **1988**, *27*, 5; b. F. Kreppelt, PhD thesis, Eidgenössische Technische Hochschule (Zürich), **1991**.
[2] C. Kieninger, E. Deery, A. D. Lawrence, M. Podewitz, K. Wurst, E. Nemoto-Smith, F. J. Widner, J. A. Baker, S. Jockusch, C. R. Kreutz, K. R. Liedl, K. Gruber, M. J. Warren, B. Kräutler, *Angewandte Chemie International Edition* **2019**, *58*, 10756.
[3] a. M. J. S. Dewar, H. N. Schmeising, *Tetrahedron* **1959**, *5*, 166; b. R. L. Miller, P. G. Lykos, H. N. Schmeising, *Journal of the American Chemical Society* **1962**, *84*, 4623.
[4] B. Kräutler, W. Keller, M. Hughes, C. Caderas, C. Kratky, *Journal of the Chemical Society, Chemical Communications* **1987**, 1678.
[5] J. D. Dunitz, E. F. Meyer Jr., *Helvetica Chimica Acta* **1971**, *54*, 77.
[6] C. Kieninger, J. A. Baker, M. Podewitz, K. Wurst, S. Jockusch, A. D. Lawrence, E. Deery, K. Gruber, K. R. Liedl, M. J. Warren, B. Kräutler, *Angewandte Chemie International Edition* **2019**, *58*, 14568.
[7] a. V. B. Pett, M. N. Liebman, P. Murray-Rust, K. Prasad, J. P. Glusker, *J. Am. Chem. Soc.* **1987**, *109*, 3207; b. C. Kratky, B. Kräutler, in *Chemistry and Biochemistry of B_{12}* (Ed.: R. Banerjee), **1999**, pp. 9; c. B. Kräutler, R. Konrat, E. Stupperich, G. Faerber, K. Gruber, C. Kratky, *Inorg. Chem.* **1994**, *33*, 4128.
[8] a. A. D. Becke, *Physical Review A* **1988**, *38*, 3098; b. J. P. Perdew, *Physical Review B* **1986**, *33*, 8822.
[9] J. P. Perdew, K. Burke, M. Ernzerhof, *Physical Review Letters* **1996**, *77*, 3865.
[10] F. Weigend, R. Ahlrichs, *Physical Chemistry Chemical Physics* **2005**, *7*, 3297.
[11] K. Eichkorn, O. Treutler, H. Ohm, M. Haser, R. Ahlrichs, *Chemical Physics Letters* **1995**, *242*, 652.
[12] S. Grimme, J. Antony, S. Ehrlich, H. Krieg, *J. Chem. Phys.* **2010**, *132*, 154104.
[13] a. TURBOMOLE V7.2 2017, a development of University of Karlsruhe and Forschungszentrum Karlsruhe GmbH, 1989-2007, TURBOMOLE GmbH, since 2007; available from http://www.turbomole.com/; b. R. Ahlrichs, M. Bär, M. Häser, H. Horn, C. Kölmel, *Chem. Phys. Lett.* **1989**, *162*, 165-169.
[14] The PyMOL Molecular Graphics System, Version 1.8. 2015.
[15] B. Kräutler, B. Puffer, in *Handbook of Porphyrin Science, Vol. 25* (Ed.: K. M. Kadish, Smith, K. M., Guilard, R.), World Scientific, **2012**, pp. 133-265.
[16] F. G. Costa, J. C. Escalante-Semerena, Biochemistry **2018**, *57*: 5076-5087.
[17] A. D. Lawrence, E. Deery, K. J. McLean, A. W. Munro, R. W. Pickersgill, S. E. Rigby, M. J. Warren. *Journal of Biological Chem*istry **2008**, *283*: 10813-21.
